# Hidden asymptomatic breakthrough infection in a proof-of-concept longitudinal study on SARS-CoV-2 vaccine recipients

**DOI:** 10.1186/s12929-026-01276-5

**Published:** 2026-07-22

**Authors:** Chiaho Shih, You-Zhen Liao, Che-Yu Hsu, Ying-Chen Tsai, Ming-Yen Lin, Hsin-Ying Clair Chiou, Wen-Hui Kuan, Chih-Hsu Chang, An-Ting Liou, Yu-Chi Chou, Jih-Jin Tsai, Ping-Chang Lin, Ming-Lung Yu, Wan-Long Chuang, Jia-Jung Lee, Jer-Ming Chang, Shang-Jyh Hwang, Justin Shih, Wen-Chun Hung, Ming-Feng Hou, Inn-Wen Chong, Yuh-Jyh Jong, Jung-San Chang

**Affiliations:** 1 Graduate Institute of Medicine, Graduate Institute of Clinical Medicine, Kaohsiung Medical University Hospital, Kaohsiung Medical University, Kaohsiung, 807 Taiwan; 2 Institute of Biomedical Sciences, Academia Sinica, Taipei, 112 Taiwan; 3 Division of Nephrology, Department of Internal Medicine, Kaohsiung Medical University Hospital, Kaohsiung Medical University, Kaohsiung, 807 Taiwan; 4 Precision Sports Medicine and Health Promotion Center, Kaohsiung Medical University, Kaohsiung, 807 Taiwan; 5 Genomics and Proteomics Core Laboratory, Kaohsiung Chang Gung Memorial Hospital, Chang Gung University College of Medicine, Kaohsiung, 833 Taiwan; 6 Department of Applied Chemistry, National Chi Nan University, Nantou, Taiwan; 7 Biomedical Translation Research Center, Academia Sinica, Taipei, 11529 Taiwan; 8 Tropical Medicine Center, Kaohsiung Medical University Hospital, Kaohsiung, 807 Taiwan; 9 Division of Infectious Diseases, Department of Internal Medicine, Kaohsiung Medical University Hospital, Kaohsiung, 807 Taiwan; 10 School of Medicine, College of Medicine, Kaohsiung Medical University, Kaohsiung, 807 Taiwan; 11 Hepatobiliary Division, Department of Internal Medicine, Kaohsiung Medical University Hospital and Hepatitis Research Center, College of Medicine, Kaohsiung Medical University, Kaohsiung, 807 Taiwan; 12 Institute of Population Health Sciences, National Health Research Institutes, Zhunan, Miaoli, 350 Taiwan; 13 Independent Researcher, New York, USA; 14 National Institute of Cancer Research, National Health Research Institutes, Tainan, 704 Taiwan; 15 Department of Biomedical Science and Environmental Biology, Kaohsiung Medical University, Kaohsiung, 807 Taiwan; 16 Department of Internal Medicine and Graduate Institute of Medicine, Kaohsiung Medical University, Kaohsiung, 807 Taiwan; 17 Department of Pulmonary Medicine, Kaohsiung Medical University Hospital, Kaohsiung, 807 Taiwan; 18 Graduate Institute of Clinical Medicine, Kaohsiung Medical University; Departments of Pediatrics and Laboratory Medicine, Kaohsiung Medical University Hospital, Kaohsiung Medical University, Kaohsiung, 807 Taiwan; 19 Division of Gastroenterology, Department of Internal Medicine, Kaohsiung Medical University Hospital, Kaohsiung Medical University, Kaohsiung, 80708 Taiwan; 20 Doctoral Degree Program in Toxicology, College of Pharmacy and PhD Program in Life Science, College of Life Science, Kaohsiung Medical University, Kaohsiung, 80708 Taiwan

**Keywords:** SARS-CoV-2, COVID-19, Vaccine, Asymptomatic breakthrough infection, Anti-spike (S) neutralizing antibody (nAb), Anti-nucleocapsid (N) antibody

## Abstract

**Background:**

Individuals with asymptomatic SARS-CoV-2 infection can unknowingly transmit the virus, yet identifying such subclinical infections in post-vaccinated populations remains challenging.

**Methods:**

We conducted a longitudinal study of 129 infection-naïve vaccine recipients immunized with various combinations of SARS-CoV-2 spike (S) protein vaccine platforms. Sera were collected before the first dose (*v*1), at 2 weeks (*v*7) and 6 months (*v*8) after the third dose. Taiwan’s first major COVID-19 outbreak occurred between *v*7 and *v*8. We measured anti-nucleocapsid (anti-N) and anti-S IgG antibody titers by ELISA and assessed virus-neutralizing activity using live virus and pseudovirus assays.

**Results:**

By developing an iterative serial screening method, we identified asymptomatic breakthrough (post-vaccination) infections among unconfirmed cases. Our *v*7-*v*8 paired cohort resolved into three distinct groups: confirmed cases (21%), asymptomatic breakthrough infections (17%), and uninfected subjects (62%). In normalized *v*8 sera, confirmed cases exhibited an anti-S^+++^ (high) /anti-N^+++^ (high) phenotype, while uninfected subjects showed an anti-S^+^ (low)/anti-N^+^(baseline) phenotype. Statistical analysis validated a distinct asymptomatic group characterized by an antibody profile anti-S^++^ (intermediate) /anti-N^+^ (baseline).

**Conclusions:**

This approach may enable more accurate estimates of vaccine efficacy and infection prevalence. In a spike-vaccinated population, anti-N antibody is more a potential specific marker for COVID-19 symptomatic disease than an ideal marker for SARS-CoV-2 infection. To our knowledge, this is the first preliminary report of identification of asymptomatic breakthrough infection from a well-vaccinated population using self-matched longitudinal pairs of serum samples.

**Supplementary Information:**

The online version contains supplementary material available at 10.1186/s12929-026-01276-5.

## Introduction

SARS-CoV-2 (severe acute respiratory syndrome coronavirus 2) emerged as a global human pathogen in December 2019 [1, 2]. Despite the availability of vaccines and antivirals, numerous challenges persist in controlling COVID-19 (coronavirus disease 2019) [3], including vaccine hesitancy, long COVID, breakthrough infections, and the emergence of immune escape and drug-resistant variants [4–7].

Isolating infected individuals from the community is a critical measure for containing epidemics. However, infected individuals do not always manifest clinically apparent symptoms. People with asymptomatic (subclinical) infections remain a major source of pathogen transmission [8]. Before SARS-CoV-2 vaccine is commercially available, asymptomatic infection in an unvaccinated population can be identified simply by circulating anti-spike (anti-S) antibody (Ab) [9–11]. However, in many countries with high vaccine coverage these days, every vaccinated immunocompetent individual is expected to be anti-S positive, regardless of their status of viral infection or symptoms. Although anti-nucleocapsid (anti-N) Ab is another marker for viral infection, it was not detected in the asymptomatic infection in the unvaccinated population (i.e., lack of productive anti-N seroconversion) [9–11]. In a vaccinated population, while anti-N Ab is detectable in symptomatic breakthrough (post-vaccination) infection, it remains unclear if anti-N Ab, by itself or in combination with anti-S Ab, can be used as a serological marker for asymptomatic breakthrough infection [12]. Recently, it was reported that undetected SARS-CoV-2 infections can be identified by the clustering of anti-N Ab trajectories [13]. To date, identification of asymptomatic breakthrough infection in a well-vaccinated population remains an important issue in the field. Accurate diagnosis of asymptomatic breakthrough infection would be highly valuable for epidemiological practice, enabling better estimates of prevalence rates, vaccine efficacy, and herd immunity.

Neutralizing antibodies (nAb) that recognize the SARS-CoV-2 spike (S) protein are considered the best correlate of protection [14, 15]. Spike vaccine-induced nAb decline rapidly, regardless of the vaccine platform [16]. The half-life of the protective nAb of uninfected subjects could be influenced by a third booster dose of spike mRNA vaccine [17]. Similarly, infection-acquired immunoglobulin G (IgG) to the spike protein and virus-neutralizing activity are also short-lived, declining rapidly after symptom onset [18–20]. In general, infection-induced nAb exhibit a slower decay rate than vaccine-elicited nAb [21–23]. SARS-CoV-2-specific IgG Ab in symptomatic patients decline more slowly than in asymptomatic infections [24]. In addition to anti-S IgG, anti-N IgG also declines after SARS-CoV-2 infection [25]. Comparisons between anti-N positive and negative cases reveal that waning anti-S nAb can be boosted by breakthrough infection [26]. Overall, anti-virus Ab declines over time until a new infection is encountered next time.

Here, using an iterative three-step serial screening method, we identified a distinct group with asymptomatic breakthrough infection from a 3-dose vaccine recipient cohort. An outbreak in Taiwan occurred after the third dose. Confirmed cases from this vaccinated cohort are PCR-positive or/and rapid assay (lateral flow) positive with apparent symptoms, but without hospitalization. These confirmed cases are required to officially self-report on-line to Taiwan CDC as soon as possible before access to physician-prescribed medications. Non-confirmed cases, including uninfected subjects from this 3-dose vaccinated cohort, are healthy individuals without any disease symptoms. Because non-confirmed cases are symptom-free, they had no need and no access to receive a PCR screening test. Although rapid self-test kits for viral antigens are widely available during the outbreak period, few healthy individuals would do the routine nasal swab-based self-test. Notably, asymptomatic breakthrough infections exhibited a unique phenotype characterized by a baseline anti-N Ab level and an intermediate anti-S Ab level. These findings demonstrate that undiagnosed asymptomatic breakthrough infection can be identified in vaccinated populations. Furthermore, our studies here revealed that anti-N Ab positivity is tightly associated only with disease manifestation. In contrast, the lack of anti-N Ab is not associated with the lack of viral infection in spike-vaccinees. Finally, we also discussed the potential changes of antibody decay and durability index after the booster dose (CADDIBOO) in homologous and heterologous combinations of vaccine platforms. A surge in the CADDIBOO value of anti-S Ab, without an accompanying increase of anti-N Ab, could suggest a candidate of asymptomatic breakthrough infection. Our study here may have implications for more accurate estimates of prevalence rates, vaccine efficacy, and herd immunity. Early identification of asymptomatic breakthrough infection should contribute to improved disease control.

## Results

### Study design

**Fig. 1:**
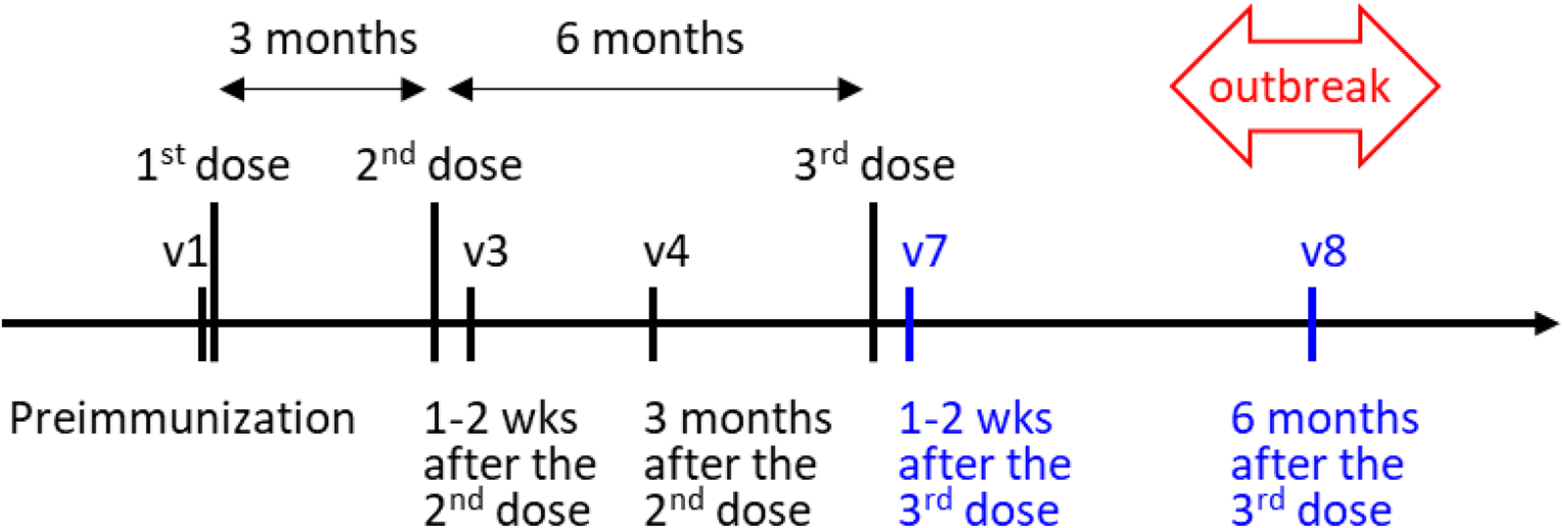
Longitudinal analysis of virus-neutralizing antibody (nAb) in SARS-CoV-2 vaccine recipients. Timeline of participant visits and serum collection at Kaohsiung Medical University Hospital (KMUH). Preimmunization sera were collected at *v*1 (1st visit) one hour before the first vaccine dose in April/May 2021. Participants primed with AZ (AstraZeneca, ChAdOx1 nCoV-19) received a 2nd dose of either AZ three months later or MO (Moderna, mRNA-1273) one to three months later. Serum samples (*v*3) were collected 1–2 weeks after the 2nd dose, and *v4* samples about three months after. A third dose with MO or BNT (Pfizer/BioNTech, BNT162b2) was given six months after the second dose. Some *v*4–*v*6 collections were discontinued when the national third dose campaign began. *v*7 sera were collected 1–2 weeks after the third dose (December 2021–March 2022), and *v*8 sera six months later. A major Omicron outbreak occurred between *v*7 and *v*8 (April-September 2022). Visit numbers in the text are mainly shown in lower case italics. To enhance visibility, visit numbers in figures and tables are sometimes shown in upper case, with or without italics

As summarized in the Introduction, it remains unclear whether asymptomatic breakthrough infection can be identified in vaccinated populations. Both anti-N and anti-S Ab naturally decline over time, regardless of whether they are induced by vaccination or natural infection [14–25]. To address this issue, we examined anti-N and anti-S Ab titers in a longitudinal cohort of 3-dose vaccine recipients immunized with combinations of different SARS-CoV-2 spike (S) vaccine platforms (Supplementary Table S1; Supplementary Fig. S1; and Methods). The timeline of vaccination and serum collection is outlined in Fig. 1. We focused on 52 self-matched pairs of sera collected at 2 weeks (7th visit, *v*7) and 6 months (*v*8) after the 3rd-dose vaccination. Between *v*7 and *v*8, Taiwan’s first major COVID-19 outbreak occurred. Confirmed cases in Taiwan are officially CDC-registered patients with respiratory symptoms and positive RT-qPCR or rapid diagnostic test results.

### Differential anti-S antibody dynamics

We compared anti-S IgG titers between *v*7 and *v*8 sera by ELISA and assessed virus-neutralizing activity using live virus and pseudovirus assays. Anti-S Ab titers measured by these assays correlated well with each other (Supplementary Fig. S2). As shown in Supplementary Fig. S3a, most sera exhibited a negative slope from *v*7 to *v*8 (upper panel, n = 52 pairs), indicating waning Ab over this 6-month period. In contrast, most confirmed cases exhibited a positive slope, indicating increasing anti-S nAb induced by recent breakthrough infection (n = 11 pairs, Supplementary Fig. S3a, middle panel). Intriguingly, some non-confirmed cases such as A3 and M31 also exhibited a positive slope (Supplementary Fig. S3a, bottom panel). When we compared the anti-S Ab titers between confirmed and non-confirmed cases in box plots using a two-sided Mann–Whitney U test, statistical significance was apparent with p-values < 0.001 (Supplementary Fig. S3b).

**Fig. 2:**
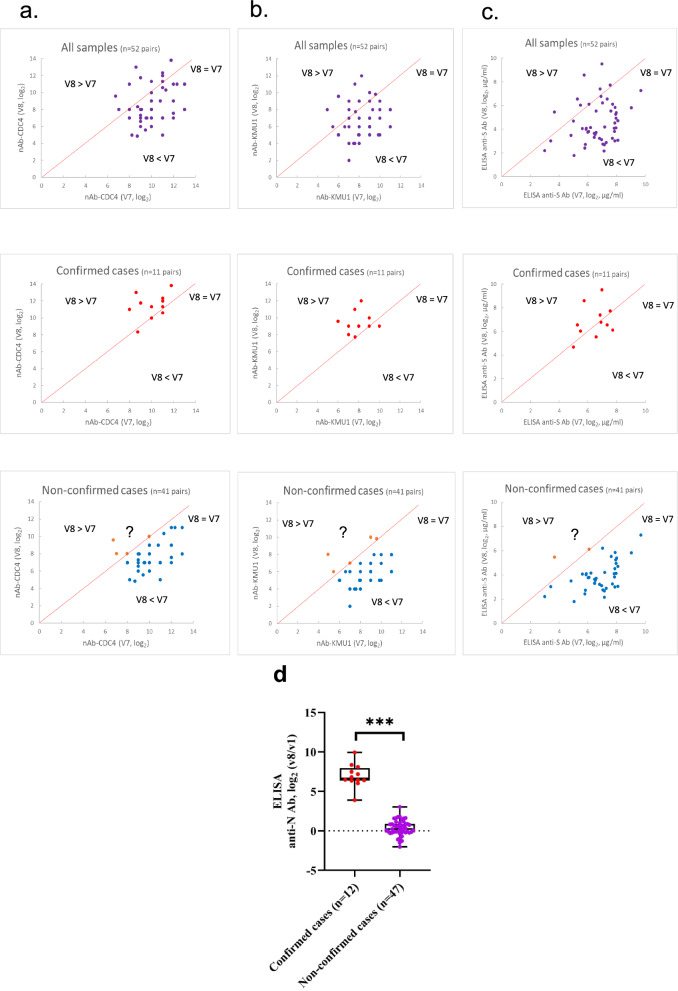
Identification of non-confirmed cases with sustained or elevated anti-S antibody titers. Serum pairs (*v7*, *v8*) from 52 participants were analyzed across assays during the Omicron outbreak (spring 2022). Anti-S antibody titers were measured by **a** live virus neutralization using the CDC4 prototype virus strain (left column); **b** neutralization with the D614G KMU1 variant strain (middle column ), and **c** ELISA (right column). Top row: all pairs (purple, *n* = 52); middle row: confirmed cases (red, *n* = 11); bottom row: non-confirmed cases (orange and blue, *n* = 41). All samples minus confirmed cases equal non-confirmed cases. Blue dots represent *v8* < *v7*; orange dots denote *v8* ≥ *v7*. Most non-confirmed cases showed declining anti-S antibody titers, though some exhibited unexplained increases (“?”). **d** Confirmed cases (*n* = 12) displayed ~ 100-fold higher *v*8/*v*1 anti-N antibody ratios than non-confirmed cases (*n* = 41) (two-sided Mann–Whitney *U* test, ***p < 0.001; median, line; IQR, box). The variation of pre-vaccinated *v*1 sera in ELISA anti-N IgG in non-confirmed cases ranges from 3 ug/ml to 147 ug/ml (data not shown). Data represent averages of duplicate measurements from two independent experiments

**Asymptomatic breakthrough infection in non-confirmed cases?**  To investigate further the cause of positive slopes of anti-S nAb in some non-confirmed cases, we plotted anti-S Ab of *v*7 versus *v*8 (Fig. 2). The diagonal line indicates near-equal anti-S Ab titers between *v*7 and *v*8. Consistent with Supplementary Fig. S3, a subpopulation (orange dots) among non-confirmed cases (n = 41; Fig. 2, bottom row) exhibited *v*8 anti-S Ab titers higher than or equal to *v*7 samples (*v*8 ≥ *v*7). Given the long 6-month interval between *v*7 and *v*8, the anti-S nAb of non-confirmed cases should have declined to lower levels if no infection encountered during this period (i.e.,* v*7 > *v*8, blue dots in Fig. 2a–c, bottom row). One potential hypothesis is that orange dot non-confirmed cases represent asymptomatic breakthrough infections.

### Confirmed cases exhibited a near 100-fold higher level of anti-N IgG

Anti-N Ab is a biomarker for SARS-CoV-2 infection [27–30]. We examined ELISA anti-N IgG in all *v*8 samples (Fig. 2d). Among 13 confirmed cases, sample A25 *v*8 serum is an outlier with a tenfold lower anti-N Ab titer relative to *v*8 sera from 12 other confirmed cases. This is most likely due to an earlier *v*8 sample collection (collection on day 9 after symptom onset is probably at a timing even before complete development of immune response). Confirmed cases (n = 12, excluding the A25 outlier) had an average anti-N Ab of approximately 3.4 mg/ml, which is about 81-fold higher than the 42.3 μg/ml average of non-confirmed cases (n = 47). The difference in *v*1-normalized *v*8 anti-N Ab (*v*8/*v*1) between confirmed and non-confirmed cases was highly statistically significant (p < 0.001; Fig. 2d). In summary, we noted a small group among non-confirmed cases containing *v*8 ≥ *v*7 anti-S Ab (Fig. 2a–c), yet lacking significant anti-N IgG (Fig. 2d). We hypothesized here that this small group could consist of putative asymptomatic breakthrough infection.

### Primary screening for candidate asymptomatic breakthrough infection from non-confirmed cases

**Fig. 3:**
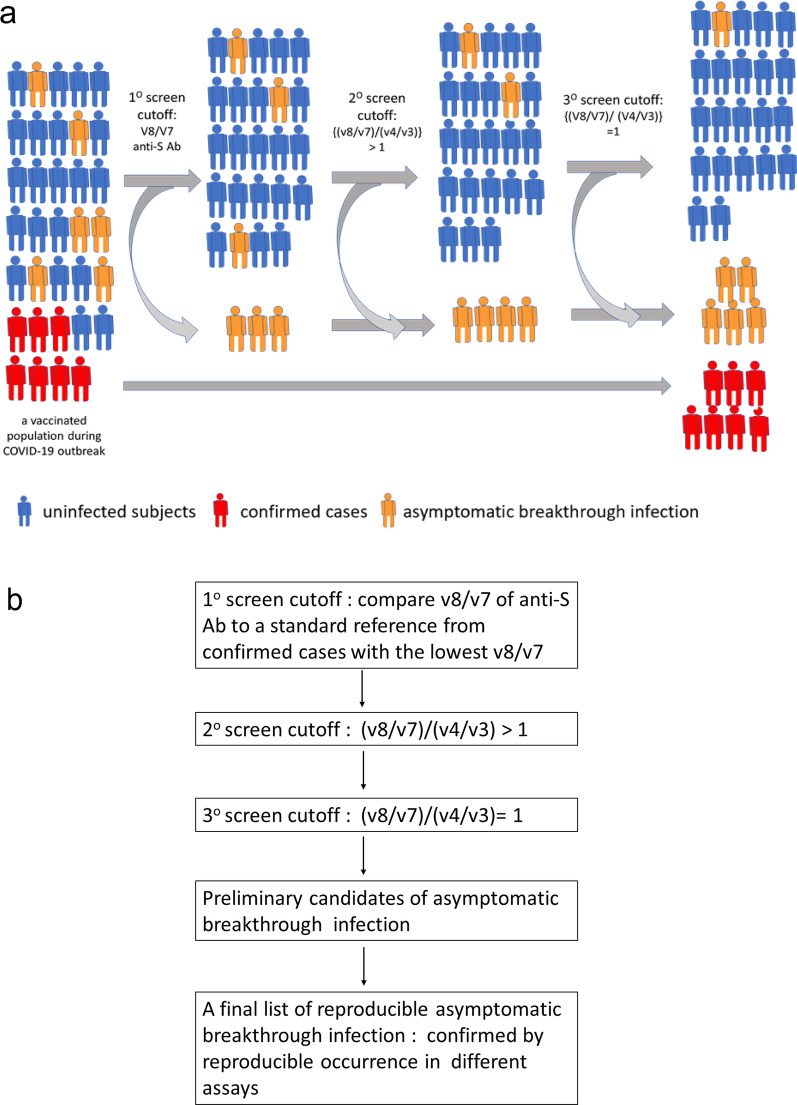

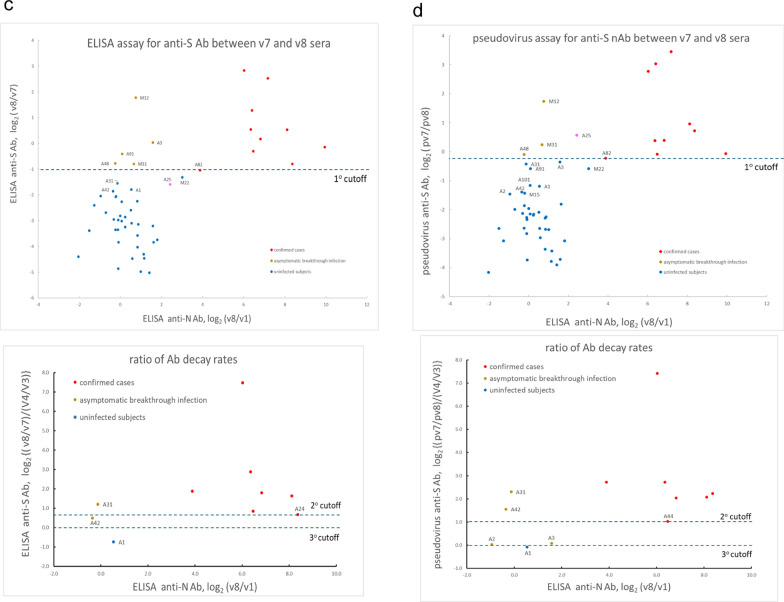

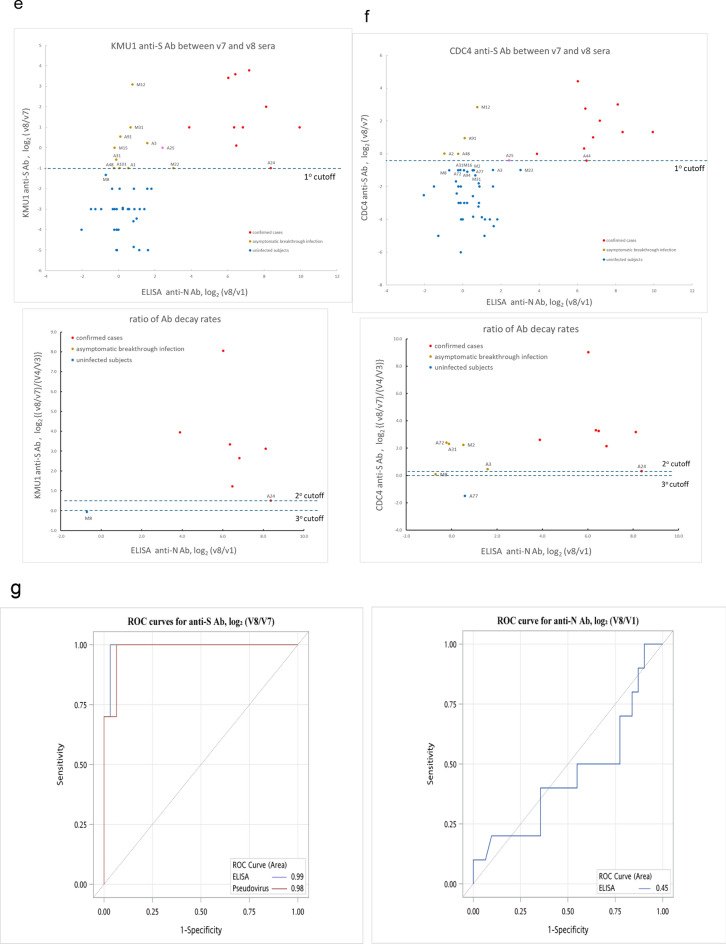
A three-step serial screening strategy for detecting potential asymptomatic SARS-CoV-2 breakthrough infections. **a** An iterative serial screening method allows the identification of asymptomatic breakthrough infection from non-confirmed cases in a vaccinated population during a COVID-19 outbreak. red: confirmed cases; yellow: asymptomatic breakthrough infection; blue: uninfected subjects. **b** Overview of the three-step iterative screening method using self-matched longitudinal serum pairs (see four examples below). **c** Double-log plot of ELISA-measured anti-S versus anti-N antibodies in *v8* samples. The *v8*/*v7* log₂ ratio (Y-axis) and *v8*/*v1* log₂ ratio (X-axis) were calculated per participant. Confirmed cases (red) defined cutoff thresholds; A82 (lowest *v8*/*v7*) was used as the primary cutoff (dotted line). Five candidates (yellow: M12, A3, A91, A48, M31) were above the line (Table 1). Samples below the dotted line are shown in blue. Confirmed case A25 outlier in pink. Secondary screening using log₂{(*v8*/*v7*)/(*v4*/*v3*)}; confirmed case A24 provided the cutoff reference. Borderline case A31 crossed the threshold. A tertiary cutoff at log_₂_{(*v8*/*v7*)/(*v4*/*v3*)} = 0 identified A42 (Table 2). **d** Pseudovirus assay comparing anti-S (inverse RLU; *pv7*/*pv8*) to ELISA-measured anti-N antibodies. Confirmed case A82 defined the primary cutoff; A48, M12, and M31 were above it (Table 1). Secondary (A44 cutoff) and tertiary screening (*pv7*/*pv8*)/(*v4*/*v3*) = 1 identified A31, A42, A2, and A3 as candidates (Table 2). **e** Live-virus neutralization against KMU1 vs. anti-N ELISA. Using confirmed case A24 as cutoff, ten samples (A1, A3, A31, A48, A91, A101, M12, M15, M22, M31) were identified (Table 1). No additional cases passed secondary/tertiary thresholds. **f** Live-virus neutralization against CDC4 vs. anti-N ELISA. Samples A2, A48, A91, and M12 exceeded the A44 cutoff (Table 1). Further screening identified A3, A31, A72, M2, and M8 (Table 2). **g** Receiver operating characteristic (ROC) curves for predicting asymptomatic SARS-CoV-2 breakthrough infection via anti-S and anti-N antibody dynamics. ROC curves for anti-spike (anti-S) antibody responses, expressed as log₂-transformed fold change between visit 8 and visit 7 (*v*8/*v*7), measured by ELISA (blue line) and pseudovirus neutralization assay (red line). Both assays demonstrated excellent discrimination between asymptomatic breakthrough and uninfected groups, with excellent AUC values of 0.99 and 0.98, respectively (Supplementary Table S2). The normalized anti-N Ab, measured by ELISA, is expressed as log₂-transformed fold change between visit 8 and visit 1 (v8/v1). The discrimination between asymptomatic breakthrough and uninfected groups was poor, with an AUC value 0.45. The diagonal random classifier line in each panel represents the no-discrimination line (area under the curve = 0.5) (Supplementary Table S2)

To systematically define this group, we designed an iterative 3-step serial screening method to identify candidate asymptomatic breakthrough infections from non-confirmed cases (Fig. 3a, b). As shown in Fig. 3c, we plotted a two-dimensional map with a Y-axis of log₂ transformed values of ELISA *v*7-normalized *v*8 anti-S Ab titer (*v*8/*v*7) versus an X-axis of log₂ transformed values of ELISA *v*1-normalized *v*8 anti-N Ab titer (*v*8/*v*1). Anti-S IgG Ab naturally declines over time (i.e., *v*7 > *v*8) in both spike vaccine recipients and convalescent COVID-19 patients [14–18]. Therefore, candidate asymptomatic breakthrough infections can be identified from non-confirmed cases if anti-S Ab has *v*8/*v*7 ≥ 1. However, for non-confirmed cases with a *v*8/*v*7 < 1 anti-S Ab and a baseline anti-N Ab, it is less straightforward to distinguish asymptomatic breakthrough infections from uninfected subjects with a decaying anti-S Ab.

Among all confirmed cases, A82 exhibited the lowest *v*8/*v*7 ratio (0.49; Table 1). We used confirmed case A82 as a primary cutoff (threshold of positivity; dotted line) to screen for candidate asymptomatic breakthrough infections. Five candidates (A3, A48, A91, M12, and M31) had *v*8/*v*7 ratios higher than A82, i.e., above the dotted cutoff line (orange dots, Fig. 3c, upper panel). The remaining non-confirmed cases below the dotted line are shown in blue, with several borderline cases labeled with their sample identities.

**Table 1:**
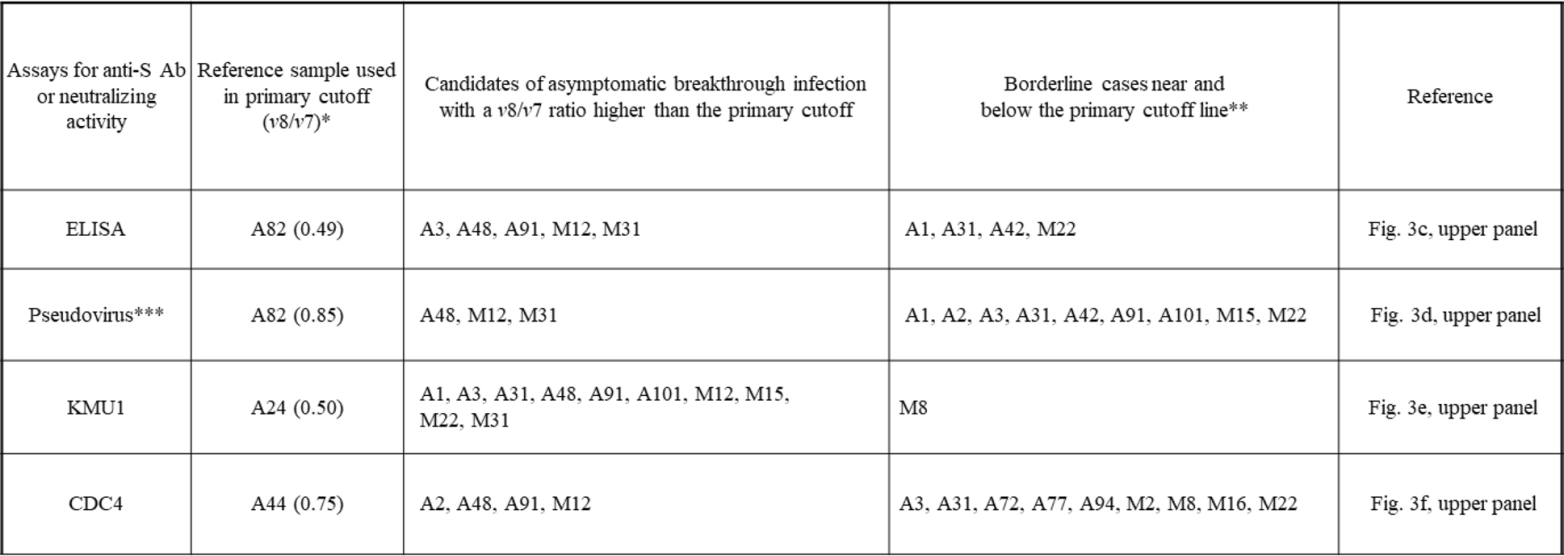
Preliminary identification of potential asymptomatic breakthrough infection from the primary screening of a three-step serial screening strategy

^*^To identify potential asymptomatic breakthrough infection, we selected a primary cutoff reference from confirmed cases solely based on its lowest value of *v*8/*v*7 in each assay. For example, in the ELISA assay, A3, A48, A91, M12, M31 are scored as candidate asymptomatic breakthrough infections because their respective *v*8/*v*7 values are larger than 0.49 of A82, i.e., above the dotted line of the primary cutoff reference A82 in the upper panels of Fig. 3c–f

^**^Borderline cases refer to those non-confirmed cases positioned near and below the primary cutoff line in the upper panels of Fig. 3c–f

^***^pseudovirus assay (RLU): p*v*7/p*v*8

### Secondary screening for more asymptomatic breakthrough infection via a secondary cutoff

To clearly differentiate slow waning of anti-S Ab in uninfected subjects from asymptomatic breakthrough infections, we used log₂ {(*v*8/*v*7)/(*v*4/*v*3)} as a secondary cutoff (Fig. 3c, lower panel). While *v*8 samples were collected 6 months after *v*7, *v*4 samples were collected 3 months after *v*3 (Fig. 1). In theory, for the same individual, the extent of reduction in anti-S Ab after 6 months should be greater than after 3 months. When we compare self-matched serum samples longitudinally-collected from the same vaccine recipient, the waning kinetics from *v*7 to *v*8 can be compared to the waning kinetics from *v*3 to *v*4 (see Discussion). For an infection-naive vaccine recipient, the *v*4/*v*3 ratio (3-month interval) is supposed to be larger than the *v*8/*v*7 ratio (6-month interval), if decay kinetics are similar between these two phases (*v3-v4* vs. *v7-v8*). However, should the ratio *v*8/*v*7 exceed *v*4/*v*3, unless the Ab decay kinetics after the third dose at v7 had changed dramatically, it could then suggest that one or more viral infections had occurred between *v*7 and *v*8 in 2022 (there was no epidemic outbreak between *v*3 and *v*4 in Taiwan in 2021). Since confirmed case A24 had the lowest {(*v*8/*v*7)/(*v*4/*v*3)} ELISA value (1.6; Table 2), A24 was chosen as the secondary cutoff to screen for additional candidate asymptomatic breakthrough infections. As shown in the lower panel of Fig. 3c, sample A31 was above the secondary cutoff line. In the third screening cycle, we further relaxed the selection stringency by using {(*v*8/*v*7)/(*v*4/*v*3)} = 1 as a cutoff, and sample A42 (orange) appeared above the dotted line of log₂ 1 = 0 (Table 2).

**Table 2:**
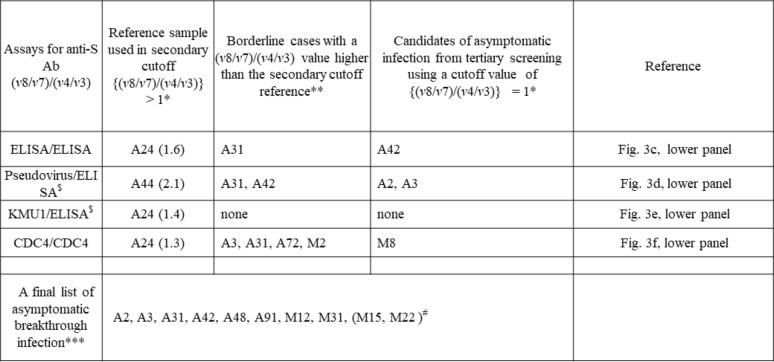
A summary of asymptomatic breakthrough infection by further screening of the borderline cases in Table 1 via a secondary and a tertiary cutoff

^*^The rationale for using (*v*8/*v*7)/(*v*4/*v*3) in further screening for asymptomatic breakthrough infection is detailed in the text

^**^New candidate asymptomatic breakthrough infection from borderline cases in Table 1 were rescreened by using (*v*8/*v*7)/(*v*4/*v*3) as a secondary cutoff

^***^These candidates are selected on the final list because they can pass the cutoff more than once in different screening assays. For example, samples A1, A72, A101, M2, and M8 were not on this final list, because they can pass the cutoff only once in only one assay

^$^The (*v*4/*v*3) data by ELISA is used here as a denominator because the ELISA data is more complete than those by pseudovirus and KMU1 assays

^#^No *v*4 samples of M15 and M22 are available for secondary and tertiary screenings. Because of their high values of (*v*8/*v*7) by the KMU1 assay in Table 1, M15 and M22 are given the benefit of doubt for inclusion on the final list

### Reproducibility by multiple assays and ROC analysis

Similar to the ELISA assay (Fig. 3c), we repeated the screening using pseudovirus (Fig. 3d) and live virus assays (Fig. 3e, f). To examine statistically the sensitivity and specificity of our screening procedures and results in Fig. 3b-f, we conducted Receiver Operating Characteristic (ROC) curve analysis for predicting asymptomatic SARS-CoV-2 breakthrough infection via anti-S (*v*8/*v*7) and anti-N (*v*8/*v*1) antibody dynamics (Fig. 3g). Both ELISA and pseudovirus assays for anti-S Ab demonstrated excellent discrimination between asymptomatic breakthrough and uninfected groups with AUC values of 0.99 and 0.98, respectively (Supplementary Table S2). In contrast, the anti-N Ab has a poor AUC value 0.45 indicating no significant difference in anti-N Ab between asymptomatic breakthrough and uninfected groups (Supplementary Table S2).

Table 1 summarizes the screening results of Fig. 3 using four different assays. Many asymptomatic breakthrough infection candidates selected from one assay were confirmed by a different assay. We compiled a final list of 10 asymptomatic breakthrough infection candidates: A2, A3, A31, A42, A48, A91, M12, M31, M15, and M22 (Table 2). One general criterion for being included on the list was to be scored positive at least twice in different assays. Because M15 and M22 lacked *v*4 serum samples, they could not be screened by the secondary cutoff {(*v*8/*v*7)/(*v*4/*v*3)}. Nevertheless, based on their consistently high *v*8/*v*7 values across different assays, M15 and M22 were included on the final list of asymptomatic breakthrough infection. In summary, using an iterative screening method across four different assays, we defined an asymptomatic breakthrough infection group (n = 10) and an uninfected group (n = 31) from non-confirmed cases.

**Fig. 4:**
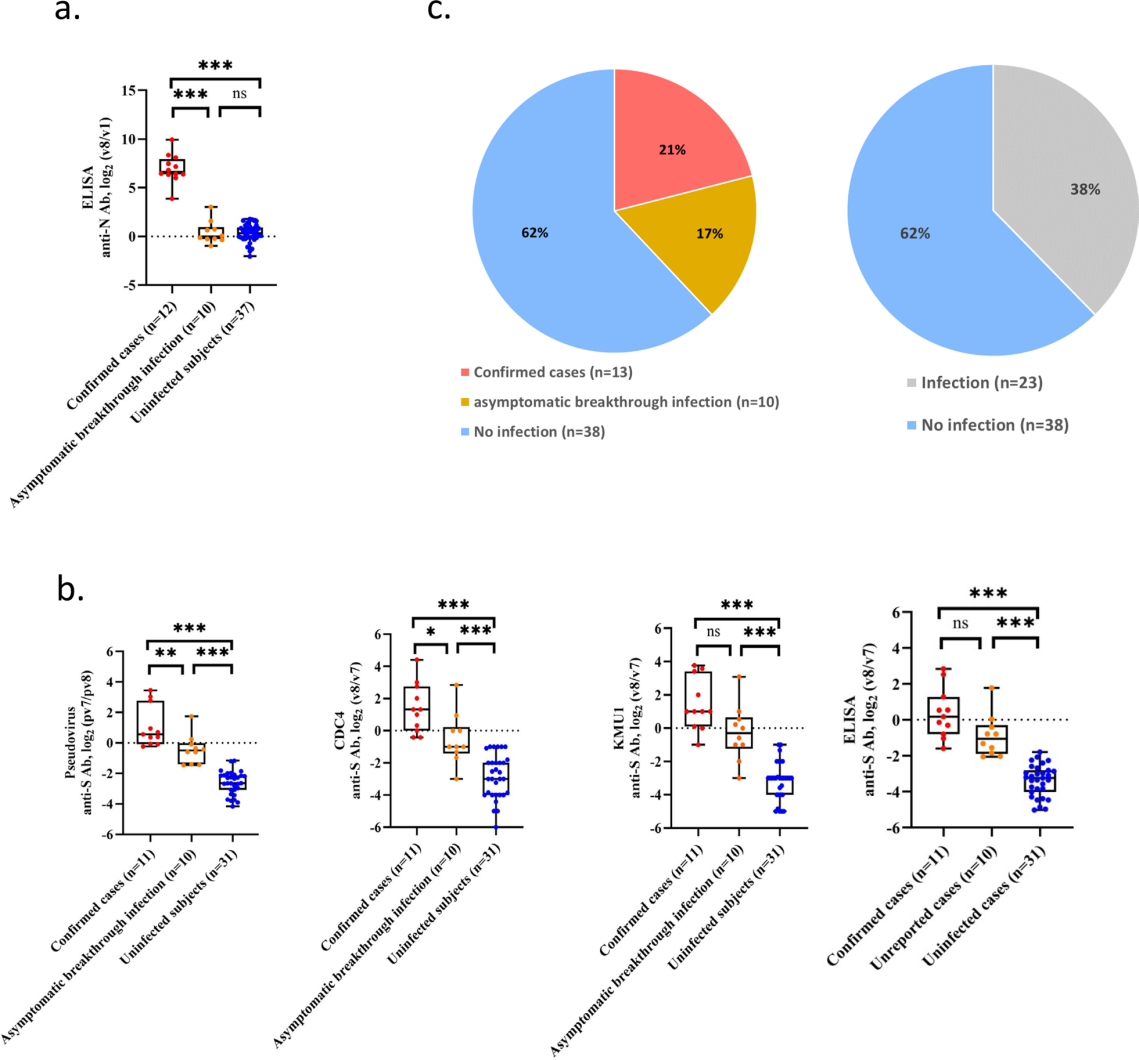
Comparison of antibody titers and infection status distributions in a three-dose vaccinated cohort. **a** Confirmed cases showed significantly higher anti-N antibody levels than asymptomatic breakthrough infection or uninfected groups (Kruskal–Wallis with two-sided Dwass–Steel–Critchlow–Fligner post-hoc test, ***p < 0.001). No difference was observed between asymptomatic breakthrough infection (*n* = 10) and uninfected subjects (*n* = 37). Dotted line: *v8* = *v1*; median (line), IQR (box). Outliers (A25, A89) were excluded. **b** Ratios of *v8*/*v7* anti-S antibody titers differed among different infection status groups. Confirmed cases and asymptomatic breakthrough infection had significantly higher ratios than uninfected subjects (***p < 0.001). Significant differences between confirmed cases and asymptomatic breakthrough infection also appeared in the pseudovirus (**p < 0.01) and CDC4 (*p < 0.05) assays. Dotted line: *v8* = *v7*; median (line), IQR (box). Unpaired confirmed cases (A15, A79) were excluded from ratio analyses (*n* = 11). **c** Estimated proportions of infection status groups in the vaccinated cohort (*n* = 61): uninfected 62%, confirmed 21%, and asymptomatic 17%. The total infected fraction is approximately 38% (grey). Two unpaired confirmed cases and seven unpaired uninfected subjects were included in the calculation

Is this newly defined group of asymptomatic breakthrough infection truly distinct from the other two infection status groups (uninfected subjects and confirmed cases)? As shown in Fig. 4a and Table 3, although confirmed cases (red) displayed nearly 100-fold higher anti-N Ab (*v*8/*v*1) than the other two groups by statistical analysis (Kruskal–Wallis method with posthoc Dwass, Steel, Critchlow-Fligner test, ***p < 0.001), there was no significant difference in *v*1-normalized anti-N Ab between asymptomatic breakthrough infection (orange) and uninfected subjects (blue). Therefore, based on our current study, despite the fact that anti-N Ab is a very sensitive and reliable marker for symptomatic infection of confirmed cases, anti-N Ab cannot, by itself, serve as a marker for asymptomatic breakthrough infection.

**Table 3:**
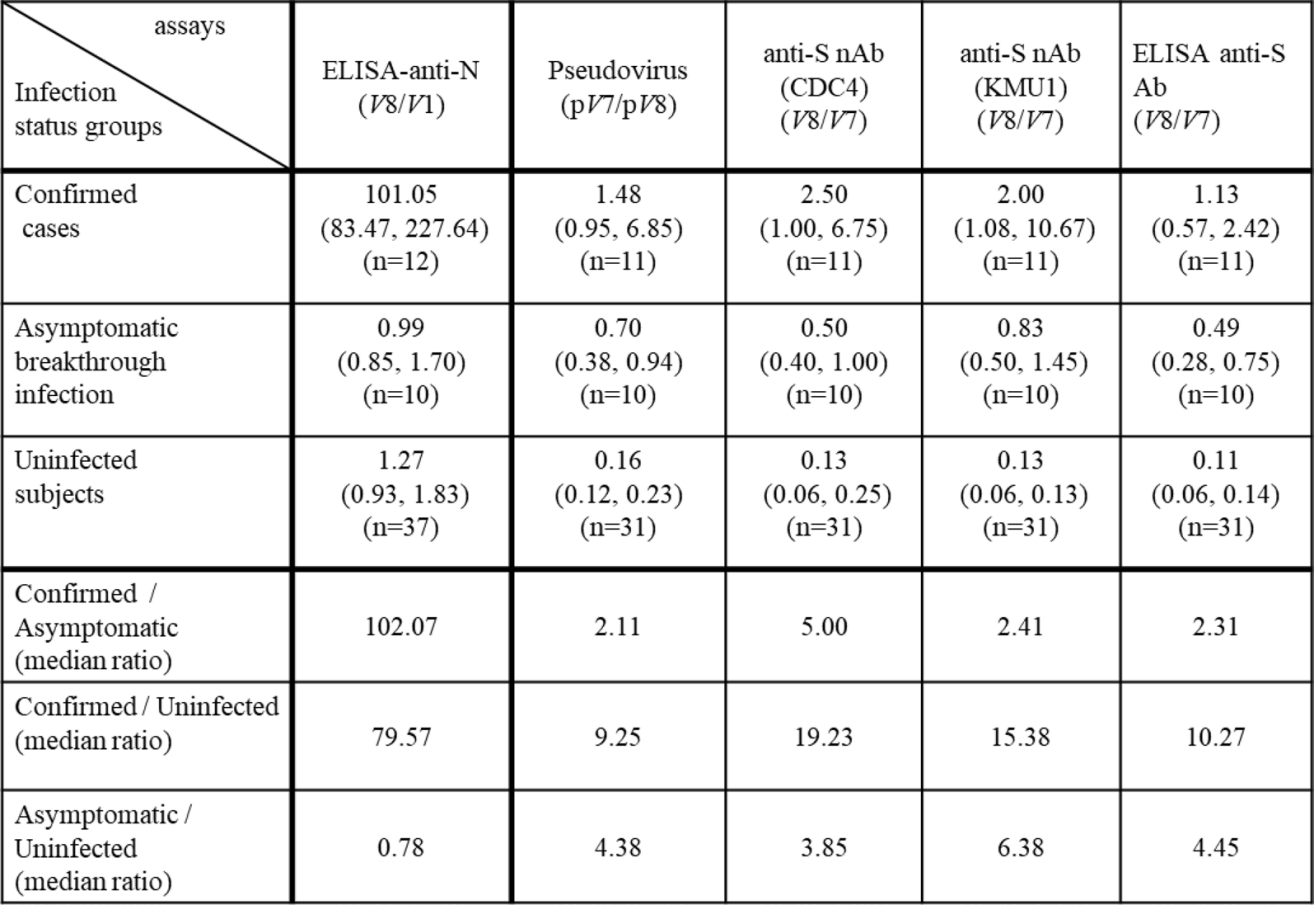
Comparision of normalized anti-S and anti-N antibody titers between different infection status group in different assays*

^*^Summary statistics of boxplots from Fig. 4a and b are presented as median and interquartile range (25th–75th percentile)

### Comparisons of antibody kinetics between different infection status groups in different assayss

Next, we compared *v*8/*v*7 ratios of anti-S Ab titers between different infection status groups (Fig. 4b and Table 3). Both confirmed cases and asymptomatic breakthrough infection exhibited significantly higher *v*8/*v*7 ratios than uninfected subjects in four out of four anti-S Ab assays (***p < 0.001). However, significant differences in *v*8/*v*7 between confirmed and asymptomatic breakthrough infection were detected only in the more sensitive pseudovirus assay (**p < 0.01) and live virus assay against the ancestral strain CDC4 (*p < 0.05), but not in the D614G variant KMU1 or the ELISA binding assays. The detected difference in *v*8/*v*7 anti-S Ab between confirmed cases and asymptomatic breakthrough infection in our vaccinated population is supported by a study using an unvaccinated population [24].

As summarized in Table 3, the median ratios of normalized anti-S Ab (*v*8/*v*7 or p*v*7/p*v*8) were approximately 10- to 20-fold between confirmed cases and uninfected subjects (9.3–19.2), approximately fivefold between asymptomatic breakthrough infection and uninfected subjects (3.9–6.4), and approximately 2- to fivefold between confirmed cases and asymptomatic breakthrough infection (2.1–5.0). Altogether, the combination of a baseline anti-N and an intermediate level of anti-S Ab titers could serve as a diagnostic marker for detecting asymptomatic breakthrough infection (anti-S^++^/anti-N^+^) in a well-vaccinated population.

### Percentages of different infection status groups in total participant populations

**Fig. 5:**
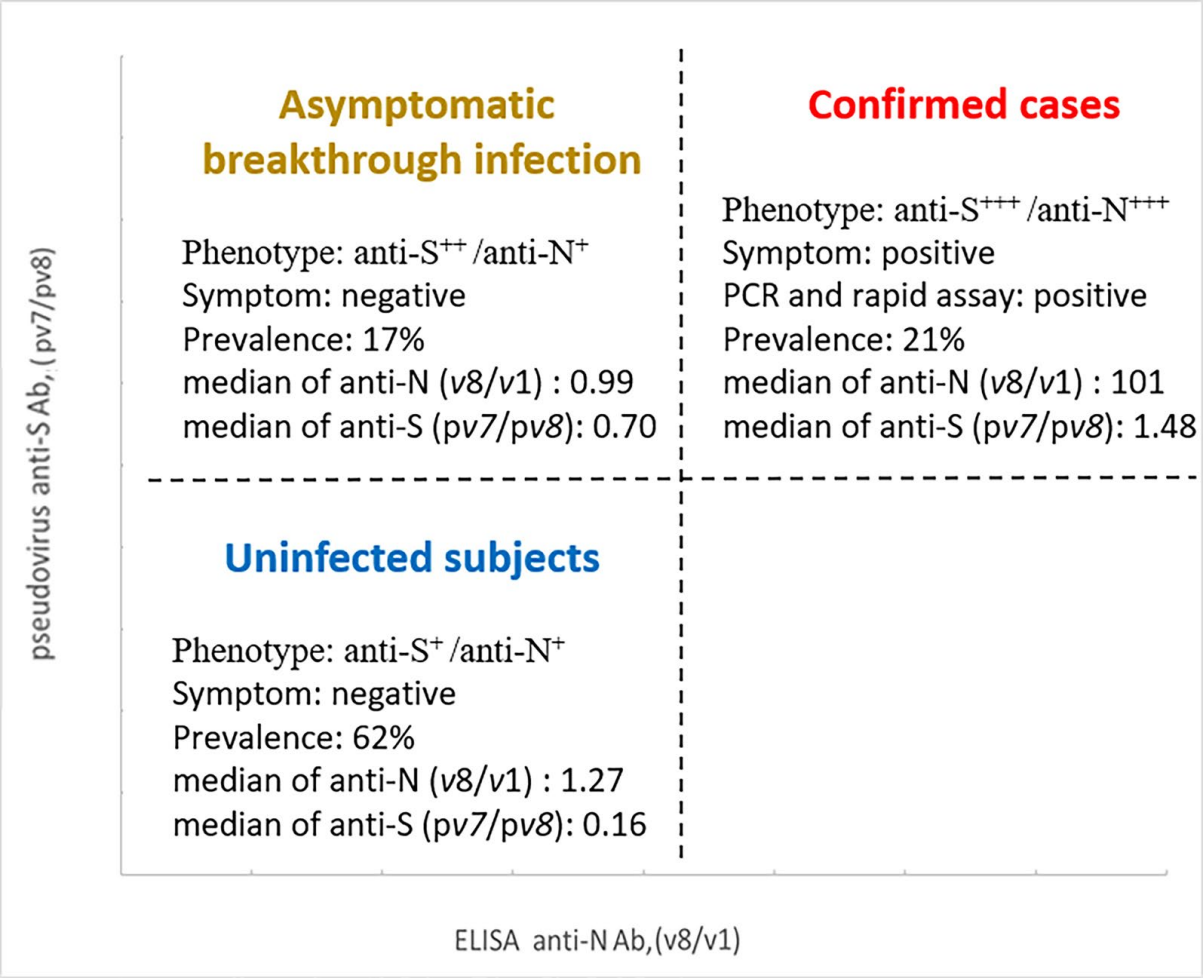
A summary of three infection status groups classified according to their serological profiles of normalized anti-N and anti-S antibody titers. A three-dose spike-vaccinated cohort (n = 61), including 52 *v*8-*v*7 pairs, was screened for asymptomatic breakthrough infection after a SARS-CoV-2 outbreak. In this diagram, all median values are taken from Table 3. Values of anti-S antibody are taken from the pseudovirus assay, as an example. Similar values of anti-S antibody titer were obtained from live virus neutralization and ELISA assays. Infection prevalence rates are from Fig. 4c. PCR test was provided only for individuals with self-reported symptoms before medication

The pie chart in Fig. 4c outlines the distribution percentages of confirmed cases, asymptomatic breakthrough infection, and uninfected subjects (21%, 17%, and 62%) in our cohort (n = 61, including unpaired samples). The total infected population was approximately 38%. None of these three infection status groups showed significant association with age, gender, comorbidities, or vaccine regimens (Fisher's Exact test, p = 0.62) (Supplementary Table S3 and Supplementary Fig. S4). Main experimental results are briefly summarized in Fig. 5.

## Discussion

In our longitudinal study using self-matched serum pairs of vaccine recipients, we designed an iterative screening method to uncover the hidden asymptomatic breakthrough infections. The cluster of asymptomatic candidates exhibited a common phenotype with intermediate anti-S Ab and baseline anti-N Ab (anti-S^++^/anti-N^+^). This phenotype is unique and distinct from symptomatic infection of confirmed cases (anti-S^+++^/anti-N^+++^) and uninfected subjects (anti-S^+^/anti-N^+^) (Figs. 4 and 5). Our Discussion Section focuses on the potential mechanisms underlying this phenotype and the potential applications and limitations of our approach to detecting asymptomatic breakthrough infection in well-vaccinated populations.

A number of possibilities could explain at least part of this anti-S^++^/anti-N^+^ phenotype: (1) An extra spike vaccine dose between *v*7 and *v*8. We consider this highly unlikely since spike vaccine supply was under the tight control by the Taiwan government, making it illegal and technically difficult to obtain an extra dose. (2) In theory, both asymptomatic breakthrough infection and the slower decay of anti-S Ab of an uninfected subject, could share a very similar anti-S^++^/anti-N^+^ phenotype. As discussed earlier, we attempted to resolve this issue by using {(*v*8/*v*7)/(*v*4/*v*3)} ≥ 1 as a cutoff in our secondary and tertiary screenings for asymptomatic breakthrough infection. Because the period of *v*8-*v*7 is twice longer than *v*4-*v*3, an uninfected subject with a slow decay anti-S Ab should have {(*v*8/*v*7)/(*v*4/*v*3)} < 1, while the *bona fide* asymptomatic breakthrough infection could have the value {(*v*8/*v*7)/(*v*4/*v*3)} ≥ 1. It is a universal phenomenon that the anti-S Ab declined soon after the third booster dose, regardless of the prime-boost regimens and nAb assay methods (Supplementary Fig. S5). In an uninfected cohort vaccinated with two doses of spike mRNA, a third booster dose of spike mRNA vaccine can decrease the decay or increase the half-life of ELISA anti-S Ab by about 1.6-fold (*t*_1/2_ ratio:100 days/60 days) [17]. In our studies, the decay kinetics of nAb from *v*7-*v*8 after the third booster dose in uninfected subjects M8 and A63, had become slower than those of *v*3-*v*4 before the third booster dose (blue and light blue lines in Supplementary Fig. S6). Furthermore, using an ELISA assay of uninfected subjects vaccinated with three doses of spike mRNA (MMM), we obtained a {(*v*8/*v*7)/(*v*4/*v*3)}value around 0.68 (Supplementary Table S4). Because the 6-month period of *v*7-*v*8 is twice longer than the 3-month period of *v*3-*v*4, the {(*v*8/*v*7)/(*v*4/*v*3)}value is supposed to be doubled (0.68 × 2 = 1.36, near 1.4), if normalized with the same length of elapsed time periods before and after the third booster dose. Therefore, despite the ethnic difference between our Taiwanese cohort and the American cohort in Roe et al. [17], the Ab decay kinetics of uninfected subjects (MMM vaccinees) were modified similarly after the third booster dose of mRNA vaccine (1.4 vs. 1.6). We coined [2 × {(v8/v7)/(v4/v3)}] values CADDIBOO (change in antibody decay and durability index after booster) in Supplementary Table S4, which includes all uninfected subjects with different vaccination platforms (MMM, AMM, AAM) and different anti-S Ab assays (CDC4, KMU1 and ELISA). Surprisingly, opposite to the CADDIBOO of AMM and MMM (both > 1), the CADDIBOO of AAM in three out of three assays is around 0.4 (less than < 1), indicating that the Ab decay kinetics after the third heterologous booster with mRNA vaccine resulted in an increased Ab decay (shorter *t*_1/2_), despite the significantly increased magnitude of Ab titer from the third booster dose in uninfected subjects of all vaccine combinations (*v*7/*v*3 =  ~ 3-fold; *v*7/*v*4 =  ~ 10-fold; Supplementary Table S5). One potential explanation could be because AAM vaccination involved a third dose “heterologous priming” (instead of a *bona fide* “homologous boost”) with a new spike mRNA. It should be clarified here that in the primary and secondary screenings in Fig. 3, we relied on certain confirmed cases as a threshold reference, rather than using any CADDIBOO from uninfected subjects as a cutoff. All in all, to cautiously exclude from the list of asymptomatic breakthrough infection any potential participants with a super-slow decay of Ab after the third booster dose, we took a more conservative than exhaustive approach to using {(*v*8*/v*7)/(*v*4/*v*3)} = 1 as a tertiary cutoff, instead of using say 0.68 as a cutoff, in our tertiary screenings for asymptomatic breakthrough infection (Table 2; Supplementary Table S4). (3) Anti-N Ab is known to have a twofold faster decay than anti-S Ab [27]. Judging from the relative levels between anti-S and anti-N IgG of asymptomatic breakthrough infection, it is highly unlikely that the anti-N Ab could have already waned off so rapidly to the baseline level. (4) Technical limits of detection sensitivity of our ELISA kit. If ELISA detection sensitivity can be further improved (current calibration range 8–128 ng/ml; Materials & Methods), it might allow a higher resolution to differentiate asymptomatic breakthrough infection from some uninfected subjects at an even lower anti-N Ab level. (5) One molecular explanation for the anti-S^+ + ^/anti-N^+^phenotype of asymptomatic breakthrough infection is that the S protein, exposed on the virion surface, may be far more immunogenic than the less exposed N protein in recognition by the host immune system. (6) These asymptomatic breakthrough infections experienced a transient and abortive infection due to lower-dose virus exposure. Unlike confirmed cases, transient SARS-CoV-2 infection during the narrow window of asymptomatic breakthrough infection is probably insufficient to mount a full-blown N protein-specific humoral immune response. Kinetically, seroconversion to anti-N Ab positivity appears to require a longer time course than anti-S Ab. In particular, spike vaccine contains only spike protein and no N protein. Upon exposure to SARS-CoV-2, the induction kinetics of anti-S nAb in the 3-dose spike-vaccinated cohort should be more acute or immediate than anti-N Ab. This result is consistent with a report showing lower plasma anti-N IgG in vaccinated than unvaccinated patients [29].

The baseline level of ELISA anti-N IgG appears to be a common phenotype in asymptomatic infection shared by both vaccinated (Fig. 4a and Table 3) and unvaccinated populations [10, 11]. In a South Korean study using unvaccinated individuals, anti-S IgG was detected by ELISA in asymptomatic infections (5/7), but no anti-N IgG was detected by ELISA (0/7) [10]. Similarly, in an unvaccinated Russian cohort, only anti-S Ab was consistently detected, and no anti-N Ab was ever detected in many asymptomatic and mild COVID-19 cases [11].

In a cross-sectional study using a vaccinated (Pfizer/BioNTech) population from Netherlands, although anti-N IgG can be found in mild and hospitalized cases of SARS-CoV-2 infection, there was no statistical significance over the cutoff in detecting asymptomatic SARS-CoV-2 infection [12]. This negative result of anti-N IgG is consistent with our current finding that asymptomatic breakthrough infection exhibited only a baseline level of *v*1-normalized anti-N^+^ Ab (*v*8/*v*1) (Fig. 4a). However, while the Netherlands cross-sectional study detected no statistical significance in anti-S Ab in their asymptomatic breakthrough infection [12], we used *v7*-normalized *v*8 sera (*v*8/*v*7) and detected here highly significant anti-S^++^ (intermediate) levels in four out of four anti-S Ab assays in Taiwanese asymptomatic breakthrough infection (Fig. 4b).

One major difference between the Netherlands report [12] and our current study is in the experimental design and data processing. In our longitudinal approach, we used self-matched longitudinal pairs of serum samples so that multiple variables, which can affect the inter-individual variability, are always normalized to its own corresponding *v*7 titer (*v*8/*v*7). For example, the immunogenetic heterogeneity between individuals in an outbred population can affect efficacy and durability of both nAb and T cell immunity in the immune response to vaccination and infection. Furthermore, anti-S IgG levels can also be influenced by vaccine regimens (Supplementary Fig. S1). In fact, without normalization to its respective *v*7 counterpart, the neutralizing activity of *v*8 serum from uninfected sample M8 is even higher than those of asymptomatic breakthrough infection A2 and A48 (Supplementary Fig. S6).

Based on this *v*7-*v*8 paired cohort of a 3-dose vaccinated population, approximately 17% are identified asymptomatic breakthrough infections (Fig. 4c). This 17% estimate is lower than those from unvaccinated cohorts, e.g., 34.9% [31] and 56% [32]. Clearly, because of the protective effect from the 3-dose vaccination, asymptomatic breakthrough infection rates in vaccinated populations should be lower than those from unvaccinated populations. One reason for the difference in estimated percentages of asymptomatic breakthrough infection could be the more conservative than exhaustive nature in our data processing. For example, some residual asymptomatic breakthrough infections could have a cutoff value of {(*v*8/*v*7)/(*v*4/*v*3)} < 1, which slipped through the filter of the third screening cycle (Fig. 3). Finally, some borderline asymptomatic individuals could not be scored reproducibly in a different assay (e.g., A1, A72, A101, M2, M8 in Table 2). While our current proof-of-concept pilot study collected sera at 3-month (*v*3-*v*4) and 6-month intervals (*v*7-*v*8), the same principle should be flexibly applicable to studies using a different length interval.

We noted that on rare occasions, results from different assays are not always parallel to each other. For example, *v*8 sample A81 had nearly fivefold higher anti-S IgG than *v*8 sample A84 (34 vs. 7 μg/ml) in the ELISA assay. However, opposite to our prediction, the anti-S nAb titer of A81 in the pseudovirus assay was about twofold lower than A84 (RLU 1010 vs. 527). While the Pearson correlation coefficient between these two different assays is very high (r = 0.8), it is not perfect (r = 1.0) (top panel, Supplementary Fig. S2c). We speculate that the neutralizing activity of some samples could involve other unknown immune components, such as anti-S-specific IgA, cytokines, and complement. None of these host factors can be measured by the IgG ELISA assay. Furthermore, the anti-S-RBD protein-based ELISA cannot detect any IgG specific for epitopes outside the RBD.

One application of our current study is to offer more accurate estimates of vaccine efficacy, herd immunity and infection prevalence rate (cumulative incidence during the epidemic period). The conventional approach tends to underestimate the overall prevalence rate. For example, the prevalence rate of SARS-CoV-2 infection should be 38% (21% + 17%; Fig. 4c). Similarly, vaccine efficacy can be evaluated by GMT titer and nAb durability, T cell immune response, or reduction rates of hospitalization and death [33–35]. These assays rely on laboratory settings of tissue culture and animal models, or risk differences in disease severity and mortality. Here, we propose an alternative indicator for vaccine efficacy using a ratio between the numbers of asymptomatic breakthrough infection and confirmed cases (e.g., 17% vs. 21% in Fig. 4c). A higher percentage of asymptomatic breakthrough infection in the total infected population (17%)/(38%) could suggest a higher protective vaccine efficacy.

It is possible that lower-dose viral exposure could be more predisposed to asymptomatic infection than higher-dose exposure. While viral dose itself may affect the rate of asymptomatic breakthrough infection in a pilot study, its influence should be diminished with a larger cohort. Finally, it remains to be tested whether our approach could be applicable to other infectious diseases, such as SARS and MERS (Middle East Respiratory Syndrome). One caveat here is that our method to identify asymptomatic breakthrough infection may not be applicable to the inactivated virus vaccines, such as Sinopharm and CoronaVac [36, 37]. These vaccine recipients might already have the anti-N Ab after vaccination.

One limitation of our current proof-of-concept study is the small sample size. Future study using an independent validation cohort will lend a strong support for our current findings. An ideal validation cohort need to be longitudinal (not cross-sectional), and include infection-naïve samples for normalization (e.g., v1).

## Conclusions

We described here a serological profile of anti-S^++^ (intermediate)/anti-N^+^ (baseline), characteristic of asymptomatic breakthrough infection in a well-vaccinated population. This serological phenotype is very distinct from confirmed cases (anti-S^+++^ (high)/anti-N^+++^ (high)) and uninfected subjects (anti-S^+^ (low)/anti-N^+^ (baseline)). In the spike-vaccinated population, serum anti-N Ab above the pre-vaccination baseline reflects recent symptom and disease. On the other hand, lack of serum anti-N Ab above the baseline reflects the lack of recent symptom and disease, but does *not* necessarily reflect the lack of recent viral infection. To the best of our knowledge, our preliminary report here is the first successful identification of asymptomatic breakthrough infection using self-matched pairs of serum samples longitudinally collected from a well-vaccinated population.

## Methods

### Study design

During the COVID-19 pandemic, a nationwide vaccination program in Taiwan was initiated in April, 2021. We enrolled a total of 129 SARS-CoV-2 vaccine-naïve participants through Kaohsiung Medical University Hospital (KMUH), Kaohsiung, Taiwan, with the approval of IRB (KMUHIRB-E(I)-20210056). Infection-naïve unvaccinated immunocompetent adults of all ages, genders, and ethnicities were eligible to participate (Supplementary Table S1). Briefly, the mean age of the total 129 participants was 36.2 years. Most were female (70.5%) and without comorbid disease (101/129, 78.3%). Sex was not considered a biological variable in this observational study.

#### Self-matched pairs of samples

These participants received three doses of spike vaccine and were followed up by telephone contact. During this 17-month prospective longitudinal study, an Omicron BA.4/BA.5 outbreak occurred in Taiwan from late April to September, 2022 (Fig. 1). By law, every confirmed case must report to Taiwan CDC and be officially registered. None of our confirmed cases were hospitalized. Antibodies naturally decline over time [14–25]. Taking advantage of this antibody waning phenomenon, we investigated the issue of asymptomatic breakthrough infection in a SARS-CoV-2 spike vaccinated population. Loss of serum collection at any stage of follow-up resulted in unpaired samples that could not be included for normalization of antibody titers in the self-matched pairwise analysis. Participants were primed and boosted with various vaccine platforms (Supplementary Fig. S1). Three different three-dose vaccination regimens included AAM (AZ/AZ/MO), AMM (AZ/MO/MO), and MMM (MO/MO/MO). A total of 52 self-matched *v*7-*v*8 pairs were available for our study.

### Laboratory assays

As detailed below, the titer of anti-S Ab was measured by the pseudovirus assay, live virus neutralization against an ancestral viral strains CDC4 and a D614G variant KMU-1, as well as a semi-quantitative ELISA S1-RBD protein binding assay. We observed high to very high Pearson correlations between different assay methods (Supplementary Fig. S2).

#### Cell lines

A Vero E6-derived cell line was used in live virus neutralization assays. This cell line was established by transfecting the plasmid pDUO2-hACE2-TMPRSS2a (InvivoGen, San Diego, CA, USA) into Vero E6, followed by selecting for hygromycin resistance and stable expression of human ACE2 and TMPRSS2. A human ACE2-expressing HEK293 cell line, designated as ACE2-HEK293 cells, was kindly provided by Dr. Yu-Chi Chou from the RNAi Core of the National Core Facility for Biopharmaceuticals at Academia Sinica, Taipei, Taiwan. The ACE2-HEK293 cells were used in the pseudovirus assay.

#### ELISA

The anti-SARS-CoV-2 N protein Human IgG ELISA kit (Proteintech Co.) has a calibration range 8–128 ng/ml. The anti-SARS-CoV-2 S-RBD (receptor-binding domain) protein Human IgG ELISA kit (Proteintech Co.) has a calibration range 6.25–200 ng/ml. The ELISA is considered a semi-quantitative assay since an internal reference with a serially diluted standard curve was always included in each plate. Each sample was assayed in duplicate in every experiment and an average of two independent experiments was performed for each sample following the vendor’s protocol.

#### Live virus neutralization

Two-fold serially diluted serum samples from vaccinees were examined for live virus-neutralizing activity in 96-well plates using a SARS-CoV-2 ancestral strain CDC4 (hCoV-19/Taiwan/4/2020; EPI_ISL_411927) and the variant strain KMU-1 (hCoV-19/Taiwan/KMU-1/2020; EPI_ISL_4169856, Clade GH, type VI). The KMU-1 spike gene contains only one single D614G mutation. The live virus neutralization assay was conducted in the biosafety level 3 facility at Kaohsiung Medical University Hospital following the approved protocol KMUHIRB-E(I)-20200013. Briefly, 50 μl of serially diluted serum samples were loaded in each well of a 96-well plate. In the BSL-III facility, an equal volume (50 μl) of CDC4 or KMU1 virus -containing DMEM (10% FBS) (40 TCID_50_/10^4^ cells/well) was mixed with the human serum samples and incubated at 37 °C for 1 h. The mixture of virus-human sera was then transferred to a 96-well plate pre-seeded 16–24 h earlier with 100 μl DMEM (10% FBS) of Vero E6 cells expressing ACE2-TMPRSS2. The culture was incubated in the BSL-III incubator at 37 °C for 48–72 h, followed by fixation with 4% formaldehyde, staining with 0.5% crystal violet, and de-staining with 5% sodium hypochlorite. The titer of neutralizing activity of serum samples was measured by the fold of maximal dilutions that could still protect Vero E6 cells from lysis.

#### Pseudovirus assay

The pseudotyped variant B.1.1.7 was from the Biomedical Translation Research Center, Academia Sinica, Taiwan (https://biotrec.sinica.edu.tw/pages/3473). This variant B.1.1.7 contains the following spike mutations, including 69–70 del, Y144 del, N501Y, A570D, D614G, P681H, T716I, S982A, D1118H. The synthetic DNA fragment encoding SARS-CoV-2 spike gene(s) was purchased from Integrated DNA Technologies (IDT) and cloned into the kpnI and EcoRI sites of the vector pcDNA^TM3.1^ ( +) (ThermoFisher Scientific). The C-terminal 18 amino acids of the spike protein was truncated to optimize the production of pseudotyped lentivirus. The pseudotyped lentivirus carrying the SARS-CoV-2 spike protein was generated by transiently transfecting HEK-293T cells with three plasmids pCMV-ΔR8.91 (an HIV-1 Gag-RT expression plasmid), pLAS2w.Fluc.Ppuro (a lentiviral vector containing a luciferase reporter gene and a puromycin resistance gene) and pcDNA3.1-nCoV-S (variant B.1.1.7 spike protein expression vector). The medium of transfected culture was harvested at 48 h and 72 h post-transfection. Cell debris was removed by centrifugation at 4000 × g for 10 min, and the supernatant was passed through a 0.45-µm syringe filter. Aliquots of the pseudotyped lentivirus were stored at − 80 °C before use. In the virus-neutralization assay, heat-inactivated human sera were serially diluted to the desired dilution and incubated with SARS-CoV-2 pseudotyped lentivirus in DMEM (10% FBS) for 1 h at 37 °C. The reporter virus titer for a positive control well (no serum added) was typically adjusted to approximately 10,000–20,000 RLU/well/96-well plate. The mixture was then inoculated with 10,000 HEK-293 T cells stably expressing human ACE2. Infected cells were continuously cultured for another 48 h before performing the luciferase assay. The expression level of luciferase was determined using ONE-Glo^™^ Luciferase Assay System (Promega). The relative luminescence unit (RLU) was detected by SpectraMax Mini. The neutralizing activity (predominantly anti-spike IgG) in the sera is inversely proportional to the RLU value. Each sample was assayed in duplicate in each experiment, and an average of two independent experiments was performed for each sample.

### Statistics

Box plots displayed distributions of anti-S and anti-N antibody titers across three COVID-19 infection status groups. Pearson correlation evaluated ELISA, live virus neutralization, and pseudovirus assays, with coefficients and 95% confidence intervals (CIs). Data were summarized as medians with interquartile ranges (IQRs). Mann–Whitney U compared ELISA anti-N titers between confirmed and non-confirmed cases. Fisher’s exact test, one-way ANOVA, and Kruskal–Wallis test with post-hoc analysis compared participants’ characteristics, vaccination regimens, and antibody titers among groups. To examine statistically the sensitivity and specificity of our screening procedures and results, we conducted ROC curve analysis for predicting asymptomatic SARS-CoV-2 breakthrough infection via anti-S (*v*8/*v*7) and anti-N (*v*8/*v*1) antibody dynamics. We evaluated the discriminative performance of antibody assays in predicting asymptomatic SARS-CoV-2 breakthrough infections using logistic regression models. Each antibody measure (e.g., anti-S and anti-N titers, expressed as log_2_-transformed values) was entered as an independent predictor in separate logistic regression models, with breakthrough infection status (yes/no) as the binary outcome. Predicted probabilities derived from the fitted models were used to construct ROC curves, and the area under the ROC curve was calculated to quantify discrimination. Optimal cut-off values were determined using Youden’s J statistic (sensitivity + specificity − 1), identifying the threshold that maximized the balance between sensitivity and specificity. Parameters including sensitivity, specificity, positive and negative prediction values, accuracy, and area under the ROC curve, were derived from the respective cut-off values. Differences in paired anti-S antibody titers from v7 to v8 across different assays were evaluated using paired t-tests and Wilcoxon signed-rank tests.Two-sided P-values < 0.05 were considered statistically significant.

## Supplementary Information

Supplementary Material 1.

## Data Availability

All data supporting the results of this study are available in this manuscript.

## Abbreviations

Anti-S: Anti-spike antibody
Anti-N: Anti-nucleocapsid antibody
AUC: Area under the curve
CADDIBOO: Change in antibody decay and durability index after booster
CI: Confidence interval
COVID-19: Coronavirus disease 2019
IQR: Interquartile range
RBD: Receptor binding domain
ROC: Receiver operating characteristic curve analysis
SARS-CoV-2: Severe acute respiratory syndrome coronavirus 2
