# Supplementary material for: Hidden asymptomatic breakthrough infection in a proof-of-concept longitudinal study on SARS-CoV-2 vaccine recipients
Source: J Biomed Sci. 2026 Jul 22;33:77. doi: 10.1186/s12929-026-01276-5 (PMC13393500; doi:10.1186/s12929-026-01276-5)
Supplement: Supplementary file 1 — Supplementary Material 1. [file 12929_2026_1276_MOESM1_ESM.pptx]

## Slide 1
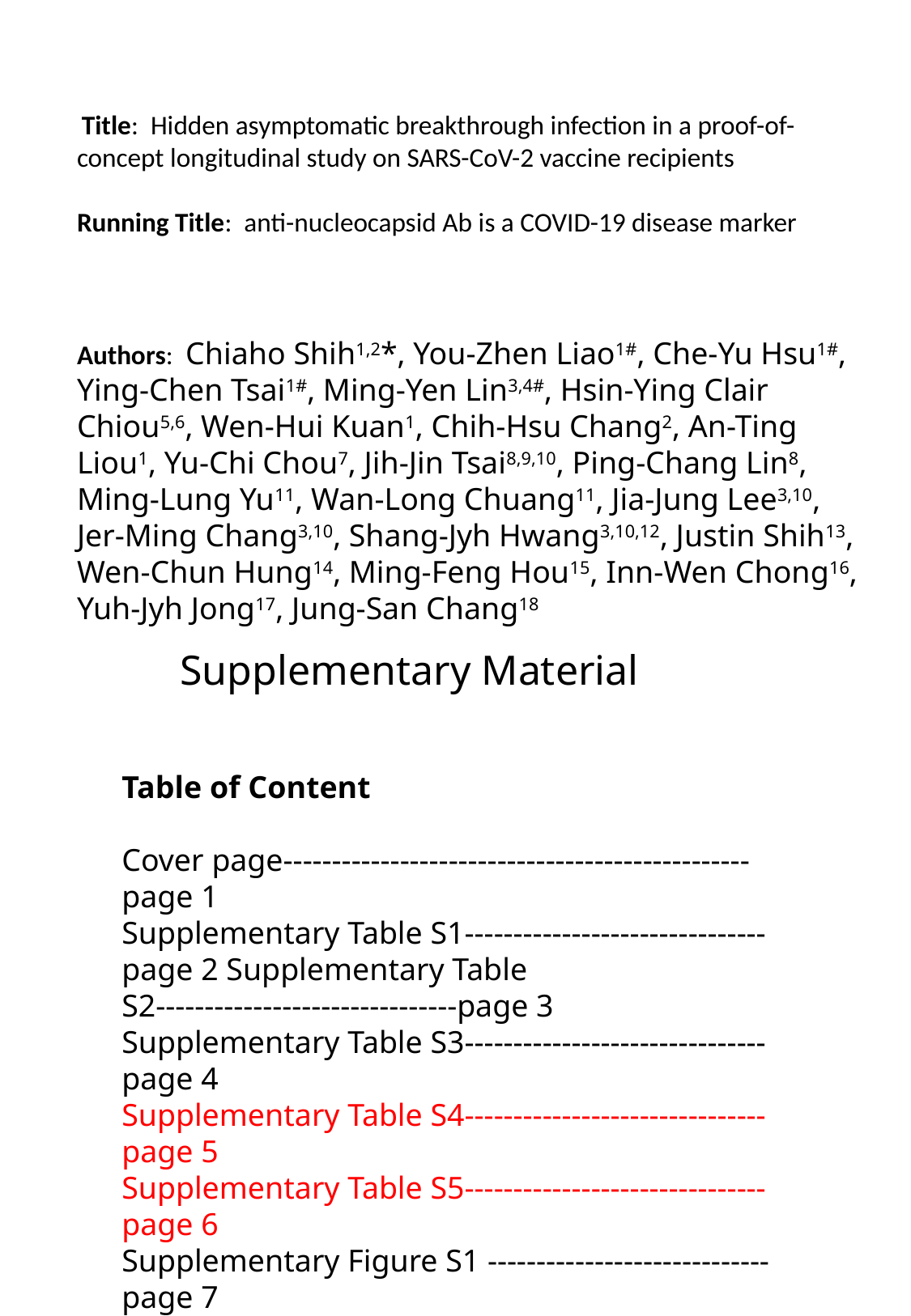

Title: Hidden asymptomatic breakthrough infection in a proof-of-concept longitudinal study on SARS-CoV-2 vaccine recipients
Running Title: anti-nucleocapsid Ab is a COVID-19 disease marker
Authors: Chiaho Shih1,2*, You-Zhen Liao1#, Che-Yu Hsu1#, Ying-Chen Tsai1#, Ming-Yen Lin3,4#, Hsin-Ying Clair Chiou5,6, Wen-Hui Kuan1, Chih-Hsu Chang2, An-Ting Liou1, Yu-Chi Chou7, Jih-Jin Tsai8,9,10, Ping-Chang Lin8, Ming-Lung Yu11, Wan-Long Chuang11, Jia-Jung Lee3,10, Jer-Ming Chang3,10, Shang-Jyh Hwang3,10,12, Justin Shih13, Wen-Chun Hung14, Ming-Feng Hou15, Inn-Wen Chong16, Yuh-Jyh Jong17, Jung-San Chang18
Supplementary Material
Table of Content
Cover page------------------------------------------------page 1
Supplementary Table S1-------------------------------page 2 Supplementary Table S2-------------------------------page 3
Supplementary Table S3-------------------------------page 4
Supplementary Table S4-------------------------------page 5
Supplementary Table S5-------------------------------page 6
Supplementary Figure S1 -----------------------------page 7
Supplementary Figure S2------------------------------page 8
Supplementary Figure S3------------------------------page 9
Supplementary Figure S4------------------------------page 10
Supplementary Figure S5------------------------------page 11
Supplementary Figure S6------------------------------page 12

## Slide 2
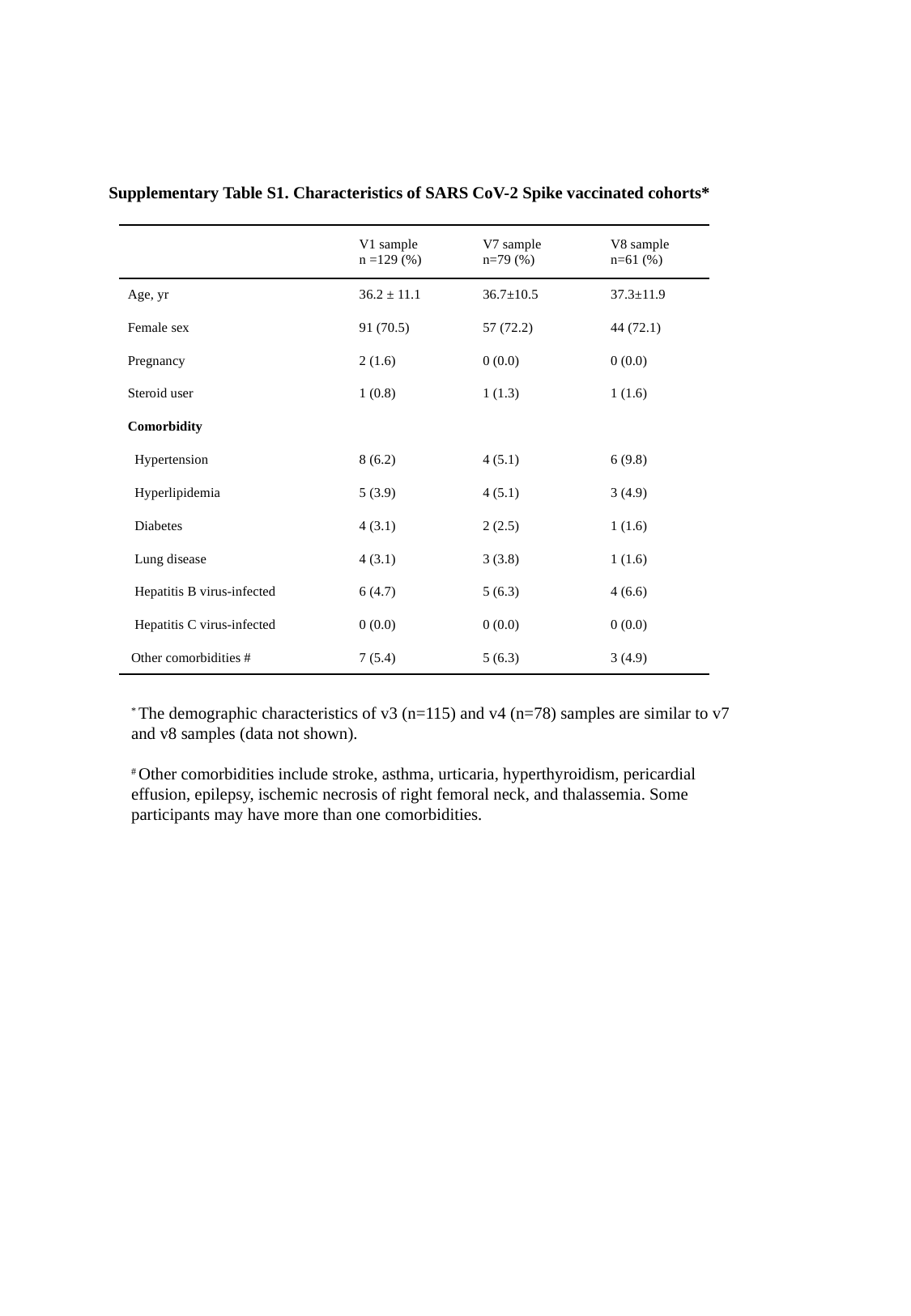

Supplementary Table S1. Characteristics of SARS CoV-2 Spike vaccinated cohorts*
| | V1 sample n =129 (%) | V7 sample n=79 (%) | V8 sample n=61 (%) |
| --- | --- | --- | --- |
| Age, yr | 36.2 ± 11.1 | 36.7±10.5 | 37.3±11.9 |
| Female sex | 91 (70.5) | 57 (72.2) | 44 (72.1) |
| Pregnancy | 2 (1.6) | 0 (0.0) | 0 (0.0) |
| Steroid user | 1 (0.8) | 1 (1.3) | 1 (1.6) |
| Comorbidity | | | |
| Hypertension | 8 (6.2) | 4 (5.1) | 6 (9.8) |
| Hyperlipidemia | 5 (3.9) | 4 (5.1) | 3 (4.9) |
| Diabetes | 4 (3.1) | 2 (2.5) | 1 (1.6) |
| Lung disease | 4 (3.1) | 3 (3.8) | 1 (1.6) |
| Hepatitis B virus-infected | 6 (4.7) | 5 (6.3) | 4 (6.6) |
| Hepatitis C virus-infected | 0 (0.0) | 0 (0.0) | 0 (0.0) |
| Other comorbidities # | 7 (5.4) | 5 (6.3) | 3 (4.9) |
* The demographic characteristics of v3 (n=115) and v4 (n=78) samples are similar to v7 and v8 samples (data not shown).
# Other comorbidities include stroke, asthma, urticaria, hyperthyroidism, pericardial effusion, epilepsy, ischemic necrosis of right femoral neck, and thalassemia. Some participants may have more than one comorbidities.

## Slide 3
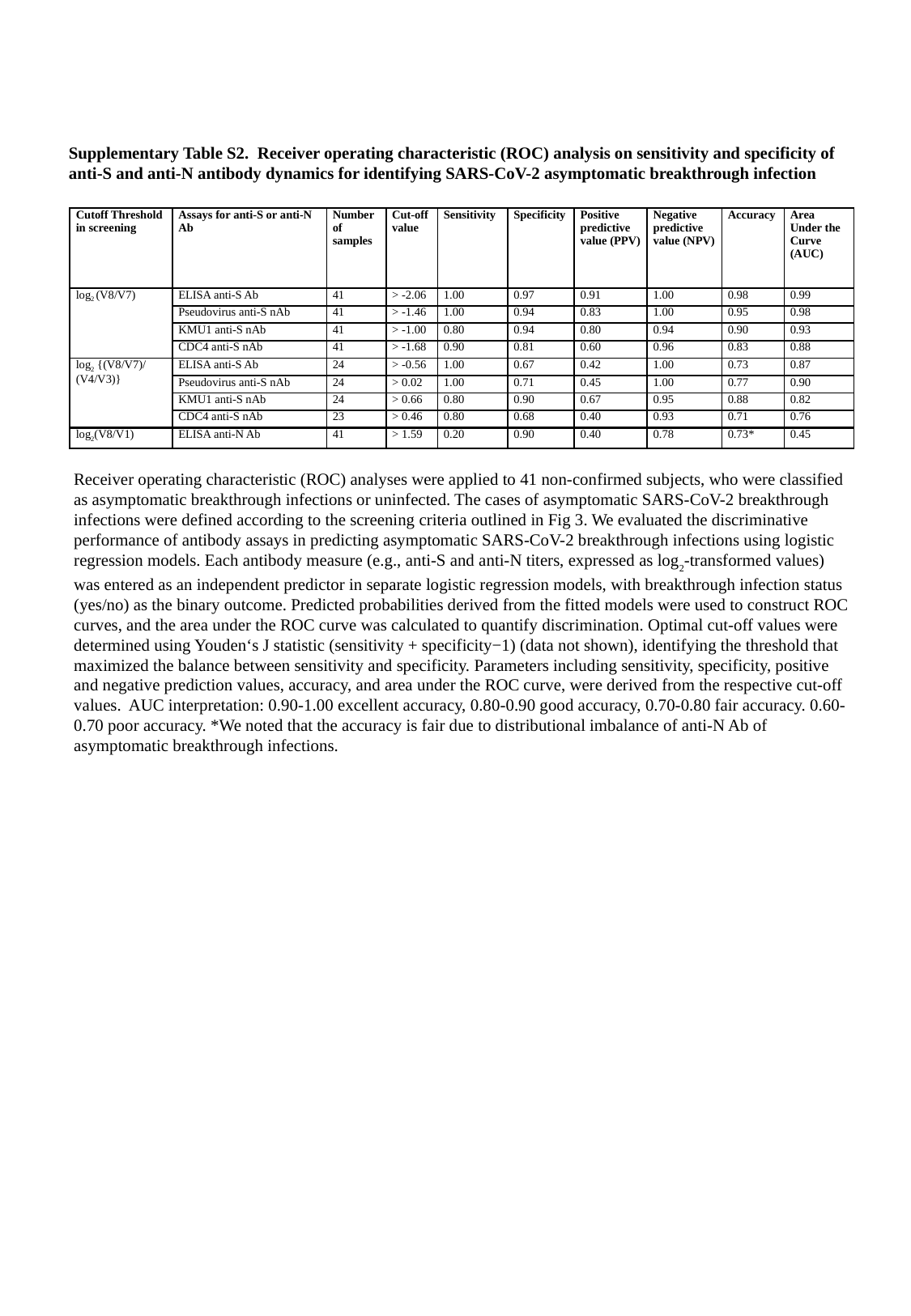

Supplementary Table S2. Receiver operating characteristic (ROC) analysis on sensitivity and specificity of anti-S and anti-N antibody dynamics for identifying SARS-CoV-2 asymptomatic breakthrough infection
| Cutoff Threshold in screening | Assays for anti-S or anti-N Ab | Number of samples | Cut-off value | Sensitivity | Specificity | Positive predictive value (PPV) | Negative predictive value (NPV) | Accuracy | Area Under the Curve (AUC) |
| --- | --- | --- | --- | --- | --- | --- | --- | --- | --- |
| log2 (V8/V7) | ELISA anti-S Ab | 41 | > -2.06 | 1.00 | 0.97 | 0.91 | 1.00 | 0.98 | 0.99 |
| | Pseudovirus anti-S nAb | 41 | > -1.46 | 1.00 | 0.94 | 0.83 | 1.00 | 0.95 | 0.98 |
| | KMU1 anti-S nAb | 41 | > -1.00 | 0.80 | 0.94 | 0.80 | 0.94 | 0.90 | 0.93 |
| | CDC4 anti-S nAb | 41 | > -1.68 | 0.90 | 0.81 | 0.60 | 0.96 | 0.83 | 0.88 |
| log2 {(V8/V7)/ (V4/V3)} | ELISA anti-S Ab | 24 | > -0.56 | 1.00 | 0.67 | 0.42 | 1.00 | 0.73 | 0.87 |
| | Pseudovirus anti-S nAb | 24 | > 0.02 | 1.00 | 0.71 | 0.45 | 1.00 | 0.77 | 0.90 |
| | KMU1 anti-S nAb | 24 | > 0.66 | 0.80 | 0.90 | 0.67 | 0.95 | 0.88 | 0.82 |
| | CDC4 anti-S nAb | 23 | > 0.46 | 0.80 | 0.68 | 0.40 | 0.93 | 0.71 | 0.76 |
| log2(V8/V1) | ELISA anti-N Ab | 41 | > 1.59 | 0.20 | 0.90 | 0.40 | 0.78 | 0.73\* | 0.45 |
Receiver operating characteristic (ROC) analyses were applied to 41 non-confirmed subjects, who were classified as asymptomatic breakthrough infections or uninfected. The cases of asymptomatic SARS-CoV-2 breakthrough infections were defined according to the screening criteria outlined in Fig 3. We evaluated the discriminative performance of antibody assays in predicting asymptomatic SARS-CoV-2 breakthrough infections using logistic regression models. Each antibody measure (e.g., anti-S and anti-N titers, expressed as log2-transformed values) was entered as an independent predictor in separate logistic regression models, with breakthrough infection status (yes/no) as the binary outcome. Predicted probabilities derived from the fitted models were used to construct ROC curves, and the area under the ROC curve was calculated to quantify discrimination. Optimal cut-off values were determined using Youden‘s J statistic (sensitivity + specificity−1) (data not shown), identifying the threshold that maximized the balance between sensitivity and specificity. Parameters including sensitivity, specificity, positive and negative prediction values, accuracy, and area under the ROC curve, were derived from the respective cut-off values. AUC interpretation: 0.90-1.00 excellent accuracy, 0.80-0.90 good accuracy, 0.70-0.80 fair accuracy. 0.60-0.70 poor accuracy. *We noted that the accuracy is fair due to distributional imbalance of anti-N Ab of asymptomatic breakthrough infections.

## Slide 4
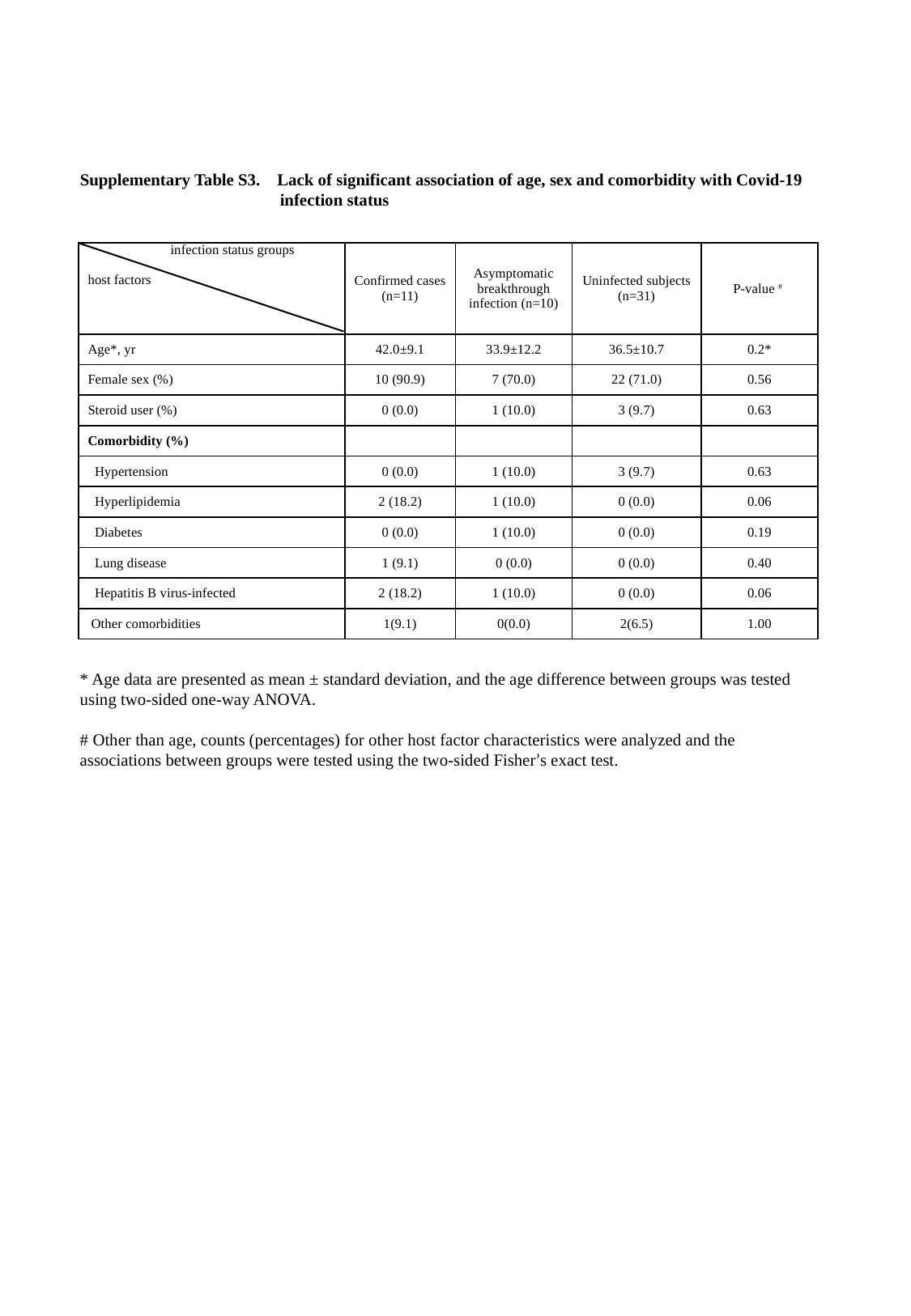

Supplementary Table S3. Lack of significant association of age, sex and comorbidity with Covid-19
 infection status
| infection status groups host factors | Confirmed cases (n=11) | Asymptomatic breakthrough infection (n=10) | Uninfected subjects (n=31) | P-value # |
| --- | --- | --- | --- | --- |
| Age\*, yr | 42.0±9.1 | 33.9±12.2 | 36.5±10.7 | 0.2\* |
| Female sex (%) | 10 (90.9) | 7 (70.0) | 22 (71.0) | 0.56 |
| Steroid user (%) | 0 (0.0) | 1 (10.0) | 3 (9.7) | 0.63 |
| Comorbidity (%) | | | | |
| Hypertension | 0 (0.0) | 1 (10.0) | 3 (9.7) | 0.63 |
| Hyperlipidemia | 2 (18.2) | 1 (10.0) | 0 (0.0) | 0.06 |
| Diabetes | 0 (0.0) | 1 (10.0) | 0 (0.0) | 0.19 |
| Lung disease | 1 (9.1) | 0 (0.0) | 0 (0.0) | 0.40 |
| Hepatitis B virus-infected | 2 (18.2) | 1 (10.0) | 0 (0.0) | 0.06 |
| Other comorbidities | 1(9.1) | 0(0.0) | 2(6.5) | 1.00 |
* Age data are presented as mean ± standard deviation, and the age difference between groups was tested using two-sided one-way ANOVA.
# Other than age, counts (percentages) for other host factor characteristics were analyzed and the associations between groups were tested using the two-sided Fisher’s exact test.

## Slide 5
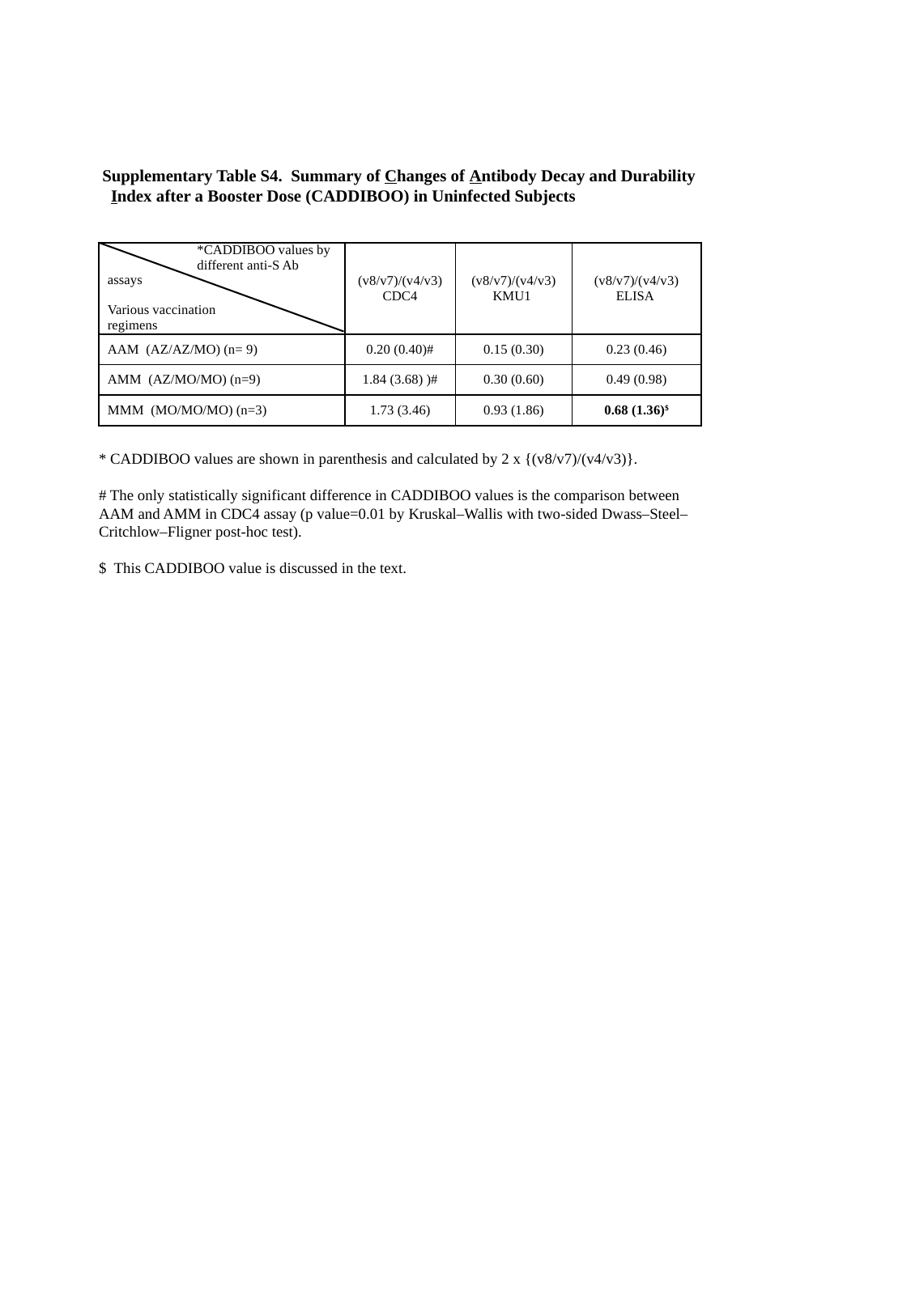

Supplementary Table S4. Summary of Changes of Antibody Decay and Durability Index after a Booster Dose (CADDIBOO) in Uninfected Subjects
| \*CADDIBOO values by different anti-S Ab assays Various vaccination regimens | (v8/v7)/(v4/v3) CDC4 | (v8/v7)/(v4/v3) KMU1 | (v8/v7)/(v4/v3) ELISA |
| --- | --- | --- | --- |
| AAM (AZ/AZ/MO) (n= 9) | 0.20 (0.40)# | 0.15 (0.30) | 0.23 (0.46) |
| AMM (AZ/MO/MO) (n=9) | 1.84 (3.68) )# | 0.30 (0.60) | 0.49 (0.98) |
| MMM (MO/MO/MO) (n=3) | 1.73 (3.46) | 0.93 (1.86) | 0.68 (1.36)$ |
* CADDIBOO values are shown in parenthesis and calculated by 2 x {(v8/v7)/(v4/v3)}.
# The only statistically significant difference in CADDIBOO values is the comparison between
AAM and AMM in CDC4 assay (p value=0.01 by Kruskal–Wallis with two-sided Dwass–Steel–Critchlow–Fligner post-hoc test).
$ This CADDIBOO value is discussed in the text.

## Slide 6
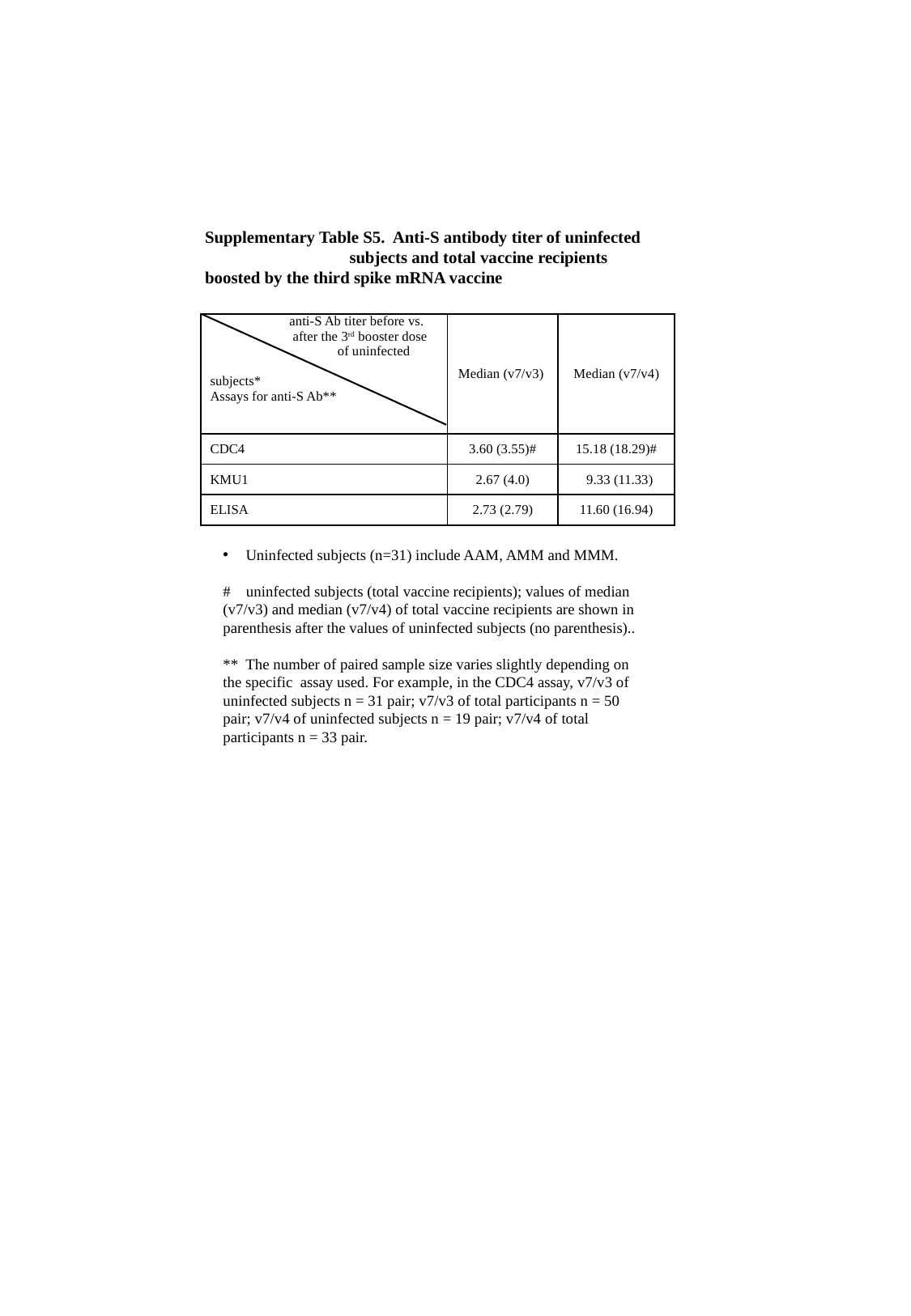

Supplementary Table S5. Anti-S antibody titer of uninfected subjects and total vaccine recipients boosted by the third spike mRNA vaccine
| anti-S Ab titer before vs. after the 3rd booster dose of uninfected subjects\* Assays for anti-S Ab\*\* | Median (v7/v3) | Median (v7/v4) |
| --- | --- | --- |
| CDC4 | 3.60 (3.55)# | 15.18 (18.29)# |
| KMU1 | 2.67 (4.0) | 9.33 (11.33) |
| ELISA | 2.73 (2.79) | 11.60 (16.94) |
Uninfected subjects (n=31) include AAM, AMM and MMM.
# uninfected subjects (total vaccine recipients); values of median (v7/v3) and median (v7/v4) of total vaccine recipients are shown in parenthesis after the values of uninfected subjects (no parenthesis)..
** The number of paired sample size varies slightly depending on the specific assay used. For example, in the CDC4 assay, v7/v3 of uninfected subjects n = 31 pair; v7/v3 of total participants n = 50 pair; v7/v4 of uninfected subjects n = 19 pair; v7/v4 of total participants n = 33 pair.

## Slide 7
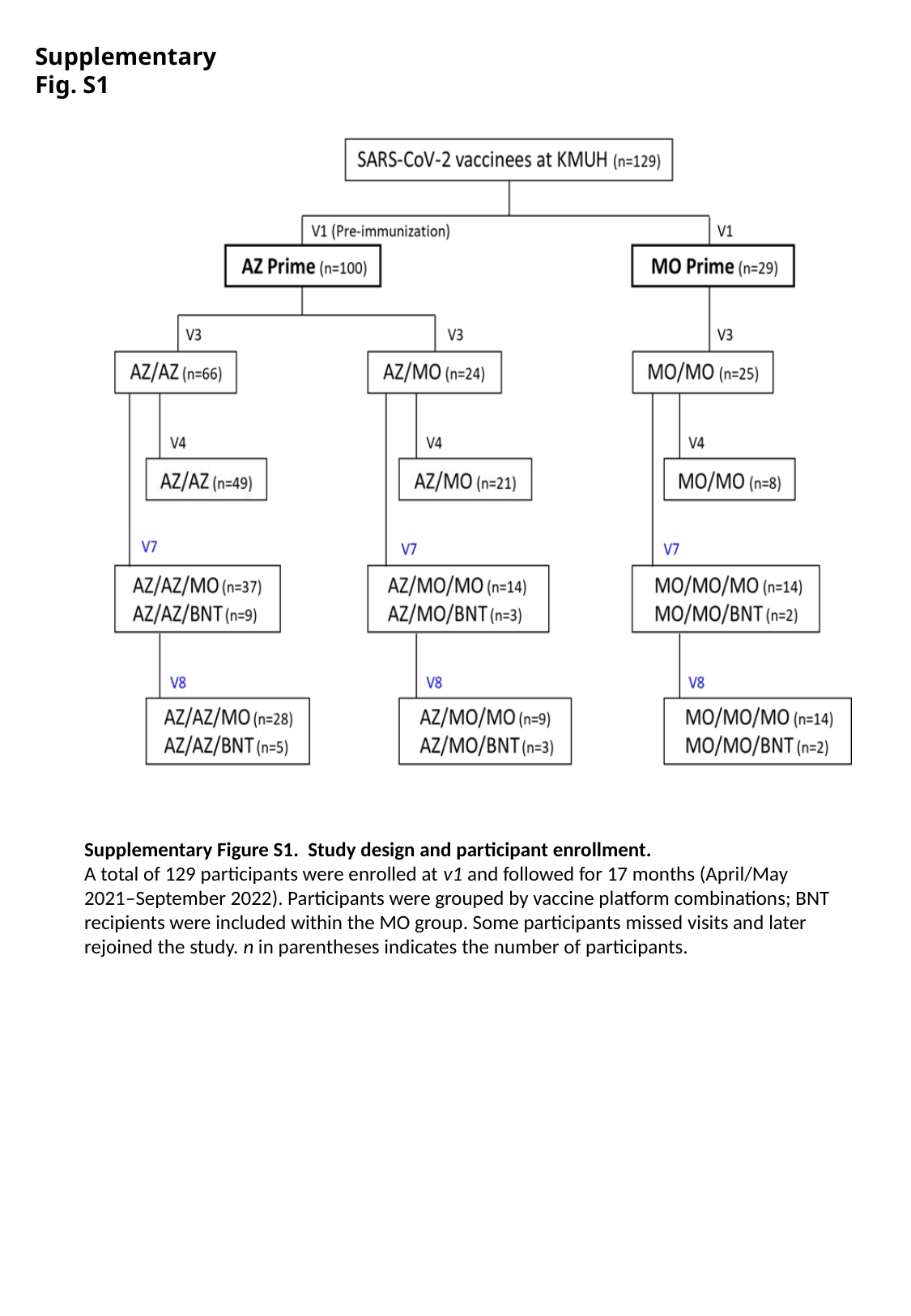

Supplementary Fig. S1
Supplementary Figure S1. Study design and participant enrollment.
A total of 129 participants were enrolled at v1 and followed for 17 months (April/May 2021–September 2022). Participants were grouped by vaccine platform combinations; BNT recipients were included within the MO group. Some participants missed visits and later rejoined the study. n in parentheses indicates the number of participants.

## Slide 8
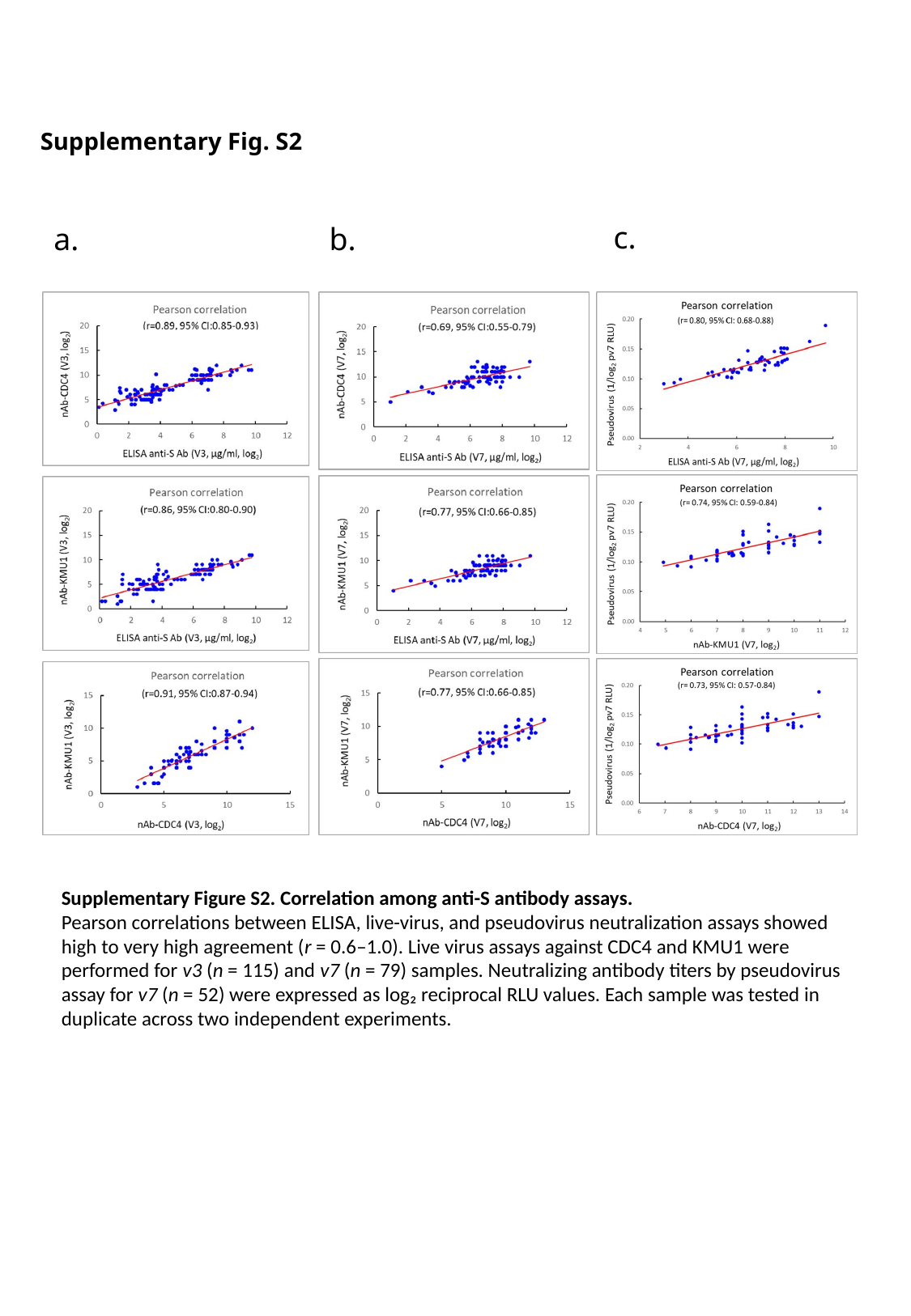

Supplementary Fig. S2
c.
a.
b.
Supplementary Figure S2. Correlation among anti-S antibody assays.
Pearson correlations between ELISA, live-virus, and pseudovirus neutralization assays showed high to very high agreement (r = 0.6–1.0). Live virus assays against CDC4 and KMU1 were performed for v3 (n = 115) and v7 (n = 79) samples. Neutralizing antibody titers by pseudovirus assay for v7 (n = 52) were expressed as log₂ reciprocal RLU values. Each sample was tested in duplicate across two independent experiments.

## Slide 9
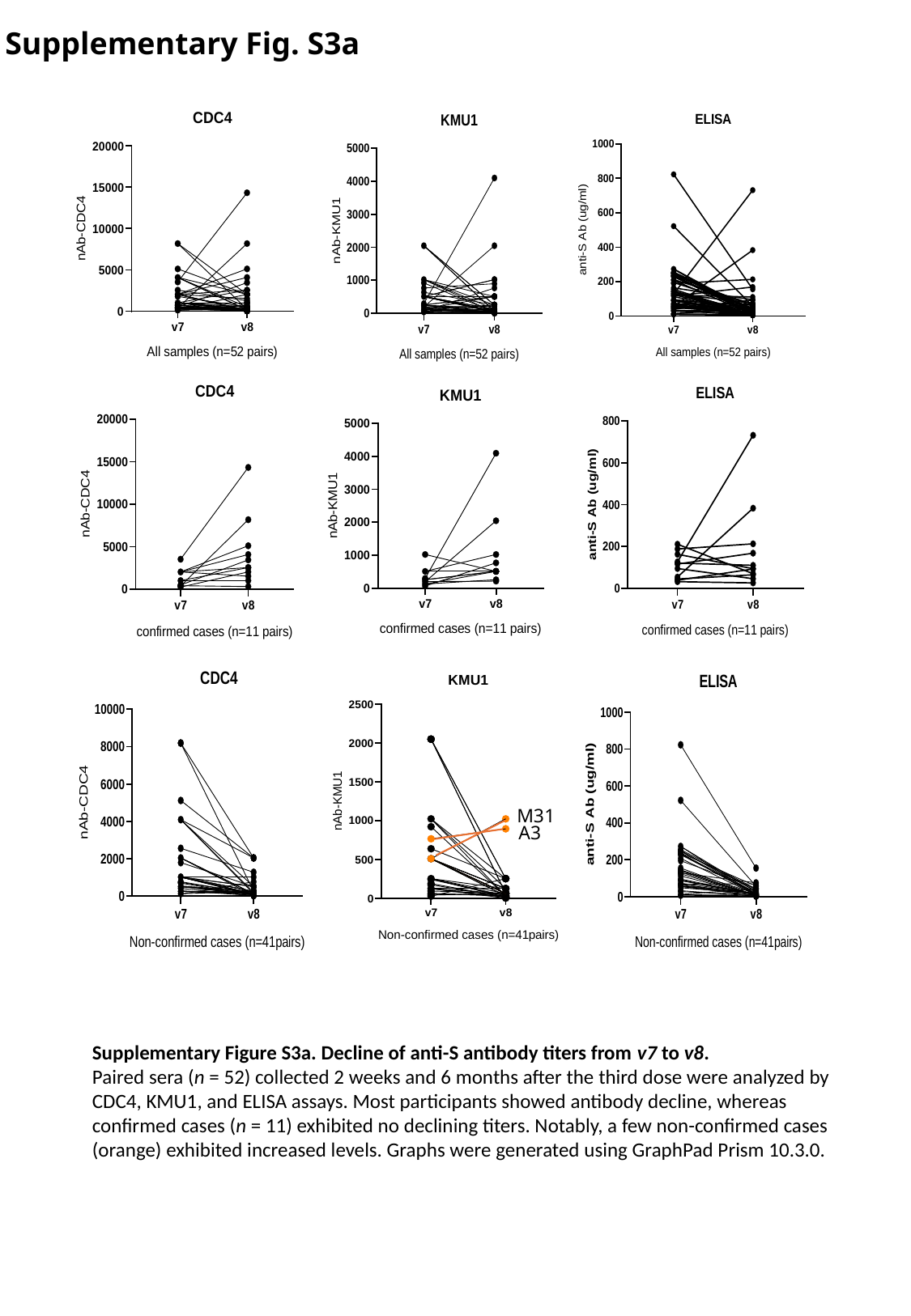

Supplementary Fig. S3a
M31
A3
Supplementary Figure S3a. Decline of anti-S antibody titers from v7 to v8.
Paired sera (n = 52) collected 2 weeks and 6 months after the third dose were analyzed by CDC4, KMU1, and ELISA assays. Most participants showed antibody decline, whereas confirmed cases (n = 11) exhibited no declining titers. Notably, a few non-confirmed cases (orange) exhibited increased levels. Graphs were generated using GraphPad Prism 10.3.0.

## Slide 10
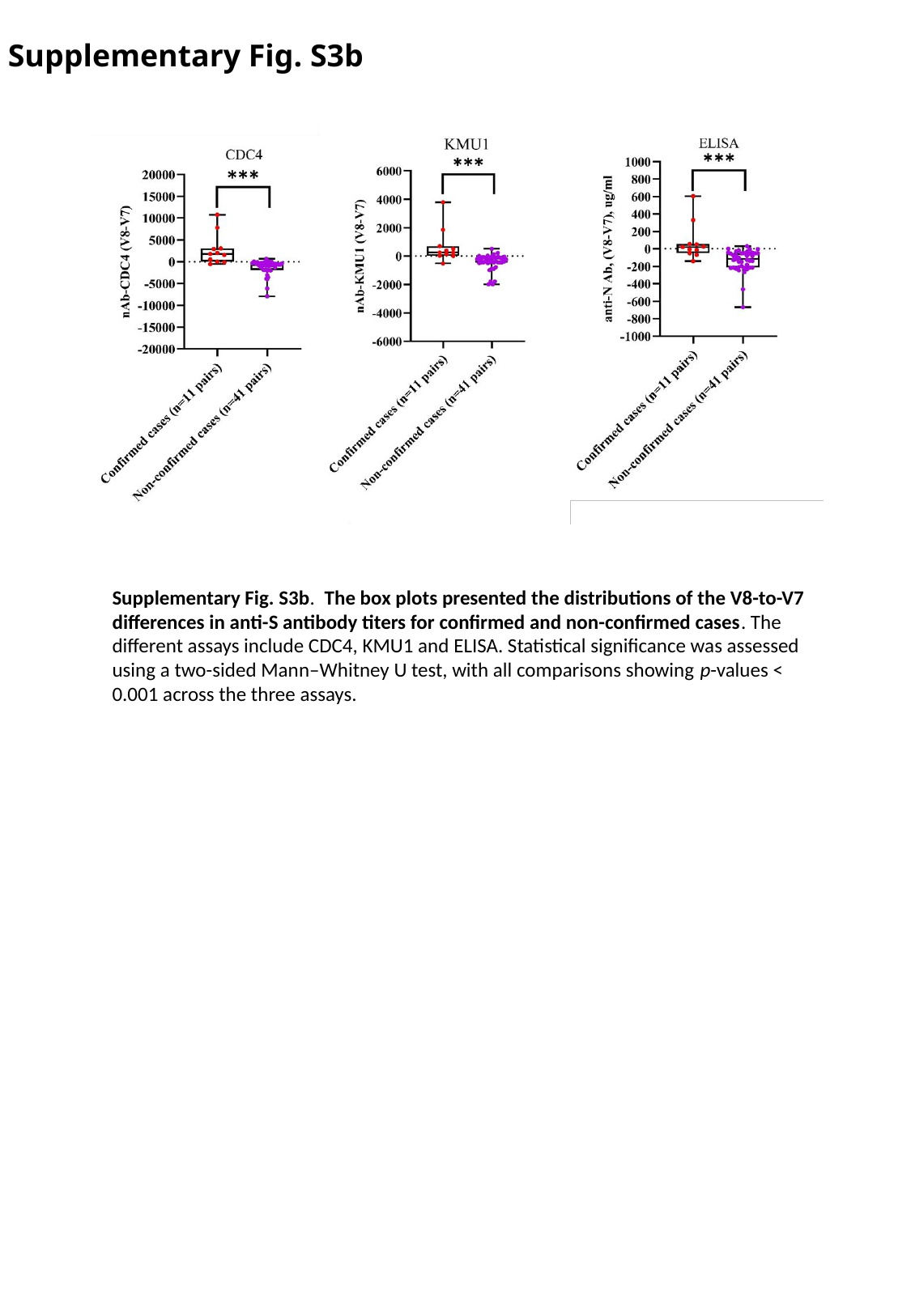

Supplementary Fig. S3b
Supplementary Fig. S3b. The box plots presented the distributions of the V8-to-V7 differences in anti-S antibody titers for confirmed and non-confirmed cases. The different assays include CDC4, KMU1 and ELISA. Statistical significance was assessed using a two-sided Mann–Whitney U test, with all comparisons showing p-values < 0.001 across the three assays.

## Slide 11
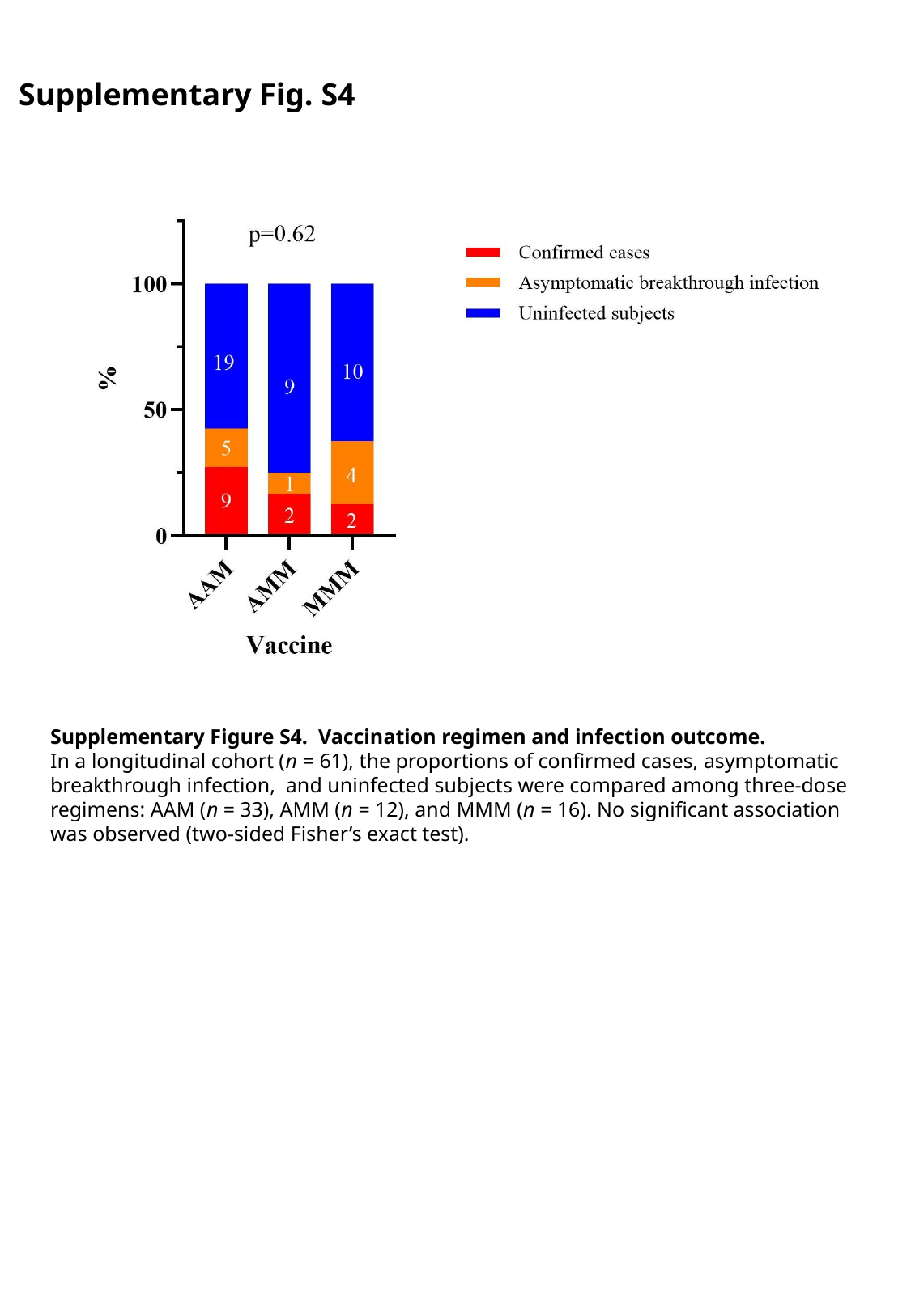

Supplementary Fig. S4
Supplementary Figure S4. Vaccination regimen and infection outcome.
In a longitudinal cohort (n = 61), the proportions of confirmed cases, asymptomatic breakthrough infection, and uninfected subjects were compared among three-dose regimens: AAM (n = 33), AMM (n = 12), and MMM (n = 16). No significant association was observed (two-sided Fisher’s exact test).

## Slide 12
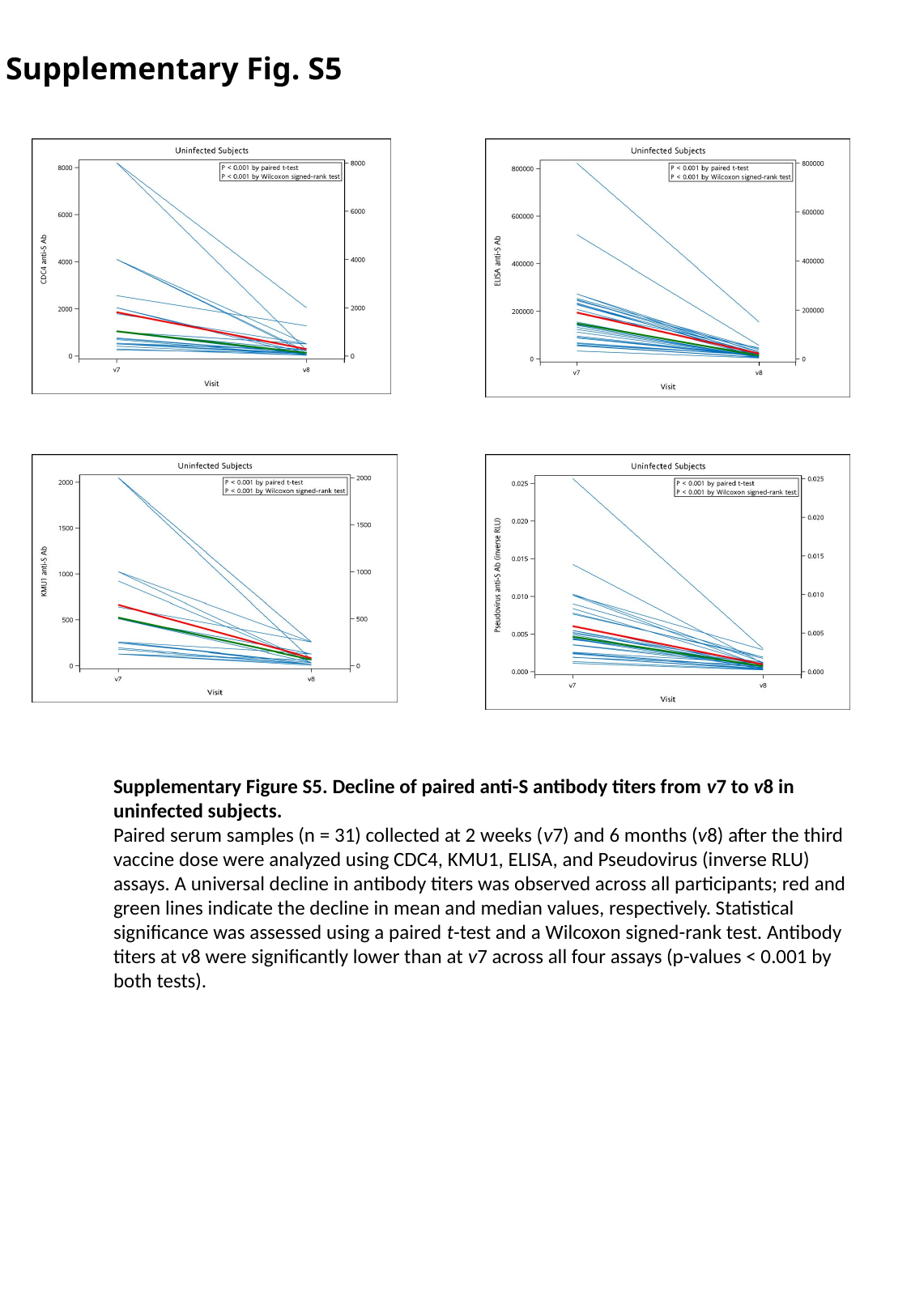

Supplementary Fig. S5
Supplementary Figure S5. Decline of paired anti-S antibody titers from v7 to v8 in uninfected subjects.
Paired serum samples (n = 31) collected at 2 weeks (v7) and 6 months (v8) after the third vaccine dose were analyzed using CDC4, KMU1, ELISA, and Pseudovirus (inverse RLU) assays. A universal decline in antibody titers was observed across all participants; red and green lines indicate the decline in mean and median values, respectively. Statistical significance was assessed using a paired t-test and a Wilcoxon signed-rank test. Antibody titers at v8 were significantly lower than at v7 across all four assays (p-values < 0.001 by both tests).

## Slide 13
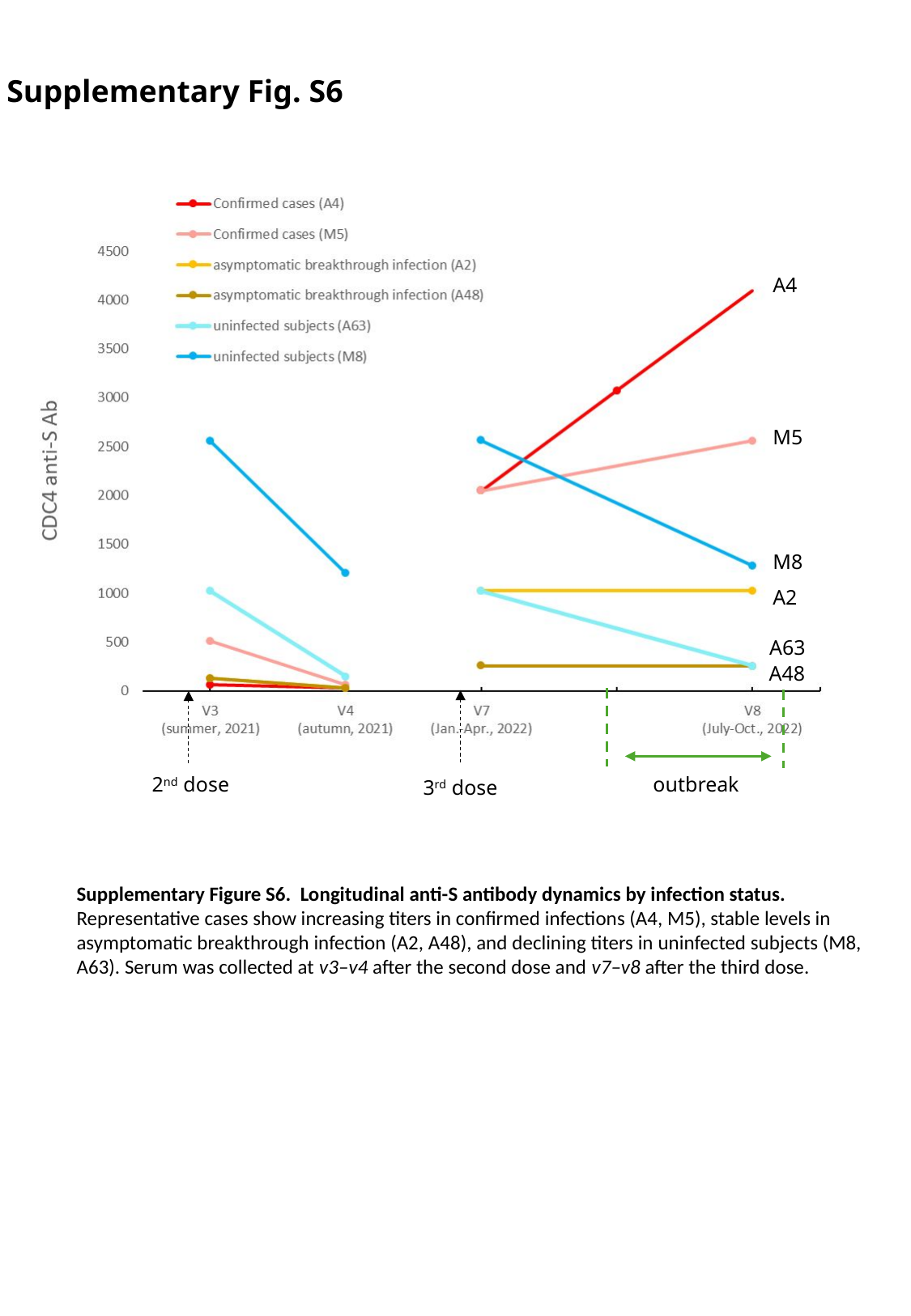

Supplementary Fig. S6
A4
M5
M8
A2
A63
A48
2nd dose
outbreak
3rd dose
Supplementary Figure S6. Longitudinal anti-S antibody dynamics by infection status.
Representative cases show increasing titers in confirmed infections (A4, M5), stable levels in asymptomatic breakthrough infection (A2, A48), and declining titers in uninfected subjects (M8, A63). Serum was collected at v3–v4 after the second dose and v7–v8 after the third dose.
